# Cigarette Smoke‐Exposed Alveolar Epithelial Cell‐Derived Exosomes Exacerbate Skeletal Muscle Dysfunction Through HDAC2 Signalling

**DOI:** 10.1002/jcsm.70349

**Published:** 2026-07-19

**Authors:** Chao Li, MingZhi Ou, Gang Jiang, GuiXian Zheng, YongLiang Jiang

**Affiliations:** ^1^ Department of Respiratory Medicine Hunan Provincial People's Hospital and the First‐Affiliated Hospital of Hunan Normal University Changsha China; ^2^ Clinical Medicine Research Center for Respiratory Rehabilitation in Hunan Province Changsha China; ^3^ Department of Obstetrics and Gynecology Changsha Hospital for Maternal and Child Health Care Changsha China; ^4^ Department of Respiratory Medicine The First Affiliated Hospital of Guangxi Medical University Nanning China

**Keywords:** COPD, crosstalk, exosomes, HDAC2, skeletal muscle dysfunction

## Abstract

**Background:**

Skeletal muscle dysfunction (SMD) is a common extrapulmonary complication of chronic obstructive pulmonary disease (COPD). Histone deacetylase 2 (HDAC2) is closely involved in the suppression of inflammatory transcription and is progressively reduced during COPD progression. Exosomes mediate intercellular communication by transferring bioactive cargos, including proteins. This study aimed to elucidate the molecular mechanism by which alveolar epithelial cell‐derived exosomes regulate HDAC2 and contribute to COPD‐related SMD.

**Methods:**

Exosome inhibitor GW4869 was used to assess the role of exosomes in skeletal muscle injury induced by chronic cigarette smoke (CS) exposure. Exosomes isolated from the bronchoalveolar lavage fluid (BALF) of CS‐exposed mice and from cigarette smoke extract (CSE)‐exposed mouse alveolar epithelial (MLE12) cells were applied to recipient mice and/or mouse myoblast (C2C12) cells to evaluate muscle phenotypes, myogenic differentiation and cellular senescence. Rescue experiments using HDAC2 overexpression or HDAC activator ITSA1 treatment, together with proteomics and protein interaction assays, were performed to elucidate the underlying molecular mechanisms.

**Results:**

GW4869 treatment ameliorated CS‐induced muscle dysfunction in mice, as evidenced by increased grip strength (222.4 ± 15.91 g vs. 159.2 ± 11.65 g, *p* < 0.001) and muscle fibre cross‐sectional area (404.0 ± 5.15 μm^2^ vs. 172.0 ± 5.39 μm^2^, *p* < 0.001), along with decreased muscle atrophy and senescence markers. In vitro, exosomes derived from 8% CSE‐exposed MLE12 cells (Exo‐CSE) impaired myogenic differentiation, decreased myotube diameter (10.50 ± 0.74 μm vs. 29.27 ± 0.48 μm, *p* < 0.001) and increased the number of senescent cells (206.7 ± 5.13 vs. 9.33 ± 1.53, *p* < 0.001). Exo‐CSE significantly reduced HDAC2 expression in C2C12 cells (0.18 ± 0.03 vs. 0.53 ± 0.04, *p* < 0.001), whereas HDAC2 overexpression or ITSA1 treatment rescued impaired myogenic differentiation and cellular senescence caused by Exo‐CSE. Proteomic analysis identified proline/arginine‐rich end leucine‐rich protein (PRELP) as a key exosomal cargo, and exosomes derived from PRELP‐silenced CSE‐exposed MLE12 cells markedly restored HDAC2 expression in recipient C2C12 cells (0.42 ± 0.02 vs. 0.18 ± 0.03, *p* < 0.001). Mechanistically, PRELP disrupted the stabilizing interaction between heat shock protein family A member 5 (HSPA5) and HDAC2, accelerating HDAC2 degradation, likely through the ubiquitin‐proteasome pathway. In vivo, the combination of PRELP knockdown and the HDAC activator ITSA1 synergistically alleviated CS‐induced muscle atrophy and senescence.

**Conclusions:**

In COPD, CS‐exposed alveolar epithelial cells release PRELP‐enriched exosomes that promote SMD by disrupting HSPA5‐mediated HDAC2 stabilization and accelerating HDAC2 degradation. Targeting the PRELP‐HDAC2 axis may represent a potential therapeutic strategy for COPD‐related SMD.

## 1 Introduction

Skeletal muscle dysfunction (SMD) is a severe extrapulmonary complication of chronic obstructive pulmonary disease (COPD), manifesting as muscle atrophy, weakness and decreased endurance [1]. It impairs physical capacity and quality of life and is associated with increased hospitalization and mortality [2]. Pathologically, COPD‐related SMD is characterized by impaired myogenic differentiation, increased protein degradation and cellular senescence [3], which together drive muscle wasting and hinder regeneration [4]. Environmental exposures, including cigarette smoke (CS) and cooking oil fumes, contribute to COPD progression and may also promote SMD [5, 6]. Despite its high clinical burden, effective therapies specifically targeting COPD‐related SMD remain limited [7], highlighting the need to elucidate its molecular mechanisms and therapeutic targets.

Exosomes are small extracellular vesicles secreted by many cell types, including alveolar epithelial cells, and contain proteins, lipids and nucleic acids [8, 9]. These vesicles deliver cargos to distal recipient cells and thereby regulate immune responses, tissue repair and disease progression and organ‐organ crosstalk in pulmonary diseases [10, 11, 12]. Skeletal muscle cells are similarly susceptible to exosomal regulation. Exosome‐derived molecules can modulate key biological processes in muscle cells, including myogenic differentiation and senescence, as shown by studies of vascular smooth muscle cell and cardiomyocyte senescence [13, 14]. Nevertheless, the role of alveolar epithelial cell‐derived exosomes in COPD‐related SMD remains largely unexplored.

In COPD, inflammatory genes are activated due to increased acetylation of core histones, which correlates with disease severity [15]. Histone deacetylases (HDACs) remove acetyl groups from histone and suppress transcription, among which HDAC2 is critical for repressing inflammatory gene expression and mediating glucocorticoid anti‐inflammatory activity [16, 17]. HDAC2 mRNA, protein expression and enzymatic activity decline progressively during COPD progression [15]. Our previous studies showed that HDAC2 inhibits skeletal muscle atrophy and senescence in CS‐induced emphysema through NF‐κB signalling [18] (Supporting Information Reference S1). Moreover, HDAC2 expression and activity can be modulated by exosomal cargos [19, 20]. However, whether epithelial exosomes regulate HDAC2 during CS‐induced COPD‐related SMD remains unknown.

In this study, we used both a CS‐induced COPD mouse model and a cell co‐culture system to investigate the role of alveolar epithelial cell‐derived exosomes in COPD‐related SMD. We demonstrated that CS‐exposed epithelial cells secreted exosomes that induced senescence and impaired differentiation in recipient myoblasts. Proteomic and functional analyses identified proline/arginine‐rich end and leucine‐rich protein (PRELP) as a key exosomal cargo. Mechanistically, exosomal PRELP disrupted the interaction of heat shock protein family A member 5 (HSPA5) and HDAC2, leading to reduced HDAC2 protein stability and myoblast dysfunction. These findings uncover a novel mechanism by which epithelial exosomes promote SMD in COPD, advancing our understanding of SMD pathogenesis and its upstream regulatory signals.

## 2 Methods

### 2.1 Study Design

This study used in vivo mouse models and in vitro mouse lung epithelial‐12 (MLE12) and murine C2C12 myoblast models. A COPD‐like mouse model was established by CS exposure, while cigarette smoke extract (CSE) was prepared to treat MLE12 cells. The exosome inhibitor GW4869 was used to investigate the role of exosomes in CS‐induced SMD. Exosomes isolated from bronchoalveolar lavage fluid (BALF) and MLE12 cells were characterized by transmission electron microscopy (TEM), nanoparticle tracking analysis (NTA) and western blot for the exosomal markers (CD63 and TSG101) and the Golgi marker (GM130). The lung‐specific marker surfactant protein C (SP‐C) was examined in BALF‐, serum‐ and muscle‐derived exosomes to assess the circulation of lung‐derived exosomes, and PKH67 fluorescence labeling was used to evaluate exosome uptake by C2C12 cells.

In vivo, body weight, grip strength and the weights of the quadriceps (Quad), gastrocnemius (Gast) and soleus (SOL) muscles were measured to evaluate muscle function. Haematoxylin and eosin (H&E) staining of gastrocnemius cross‐sections quantified myofiber cross‐sectional area (CSA) to assess muscle structure. MuRF1 and Atrogin‐1 were detected by western blot to evaluate muscle atrophy, whereas p16, p21 and p53 were detected by immunofluorescence to assess muscle senescence.

In vitro, Cell Counting Kit‐8 (CCK‐8) assays assessed C2C12 cell viability. Myosin heavy chain (MyHC) immunofluorescence staining and western blot analysis of myogenic proteins (MyHC, MyoD and MyoG) evaluated myotube formation, while senescence‐associated β‐galactosidase (SA‐β‐gal) staining and western blot analysis of p16, p21 and p53 assessed cellular senescence. CCK‐8 and terminal deoxynucleotidyl transferase dUTP nick‐end labeling (TUNEL) assays evaluated the effects of different CSE concentrations on MLE12 cell viability and apoptosis, respectively, to select an appropriate concentration for mechanistic experiments.

For mechanistic studies, proteomic analysis identified cargo proteins in exosomes from CSE‐exposed MLE12 cells. Co‐immunoprecipitation (Co‐IP) and proximity ligation assay (PLA) examined interactions among key proteins. Cycloheximide (CHX) chase assays assessed HDAC2 protein stability, and MG132 treatment determined whether HDAC2 underwent ubiquitin‐mediated degradation. Adeno‐associated virus carrying short hairpin RNA targeting PRELP (AAV‐shPRELP) was used to knock down PRELP in vivo, whereas plasmids regulated the expression of PRELP, CKAP4, NR3C1, HSPA5 and HDAC2 in vitro. The HDAC activator ITSA1 was used to pharmacologically activate HDAC2. Detailed information can be found in the Supporting Information.

### 2.2 Statistical Analysis

Data are presented as mean ± standard deviation (SD). All *n* values represent biological replicates, with *n* = 3 per group for in vitro experiments and *n* ≥ 5 per group for in vivo experiments. Analyses were performed using GraphPad Prism version 9.0. Differences between the two groups were analysed using an unpaired two‐tailed Student's *t*‐test. Multiple‐group comparisons were performed using one‐way ANOVA followed by Tukey's post hoc test, and analyses involving two independent variables were performed using two‐way ANOVA followed by Bonferroni's correction. *p* < 0.05 was considered statistically significant.

## 3 Results

### 3.1 CS Exposure Induces Skeletal Muscle Senescence and Dysfunction in Mice via Exosomes

**FIGURE 1:**
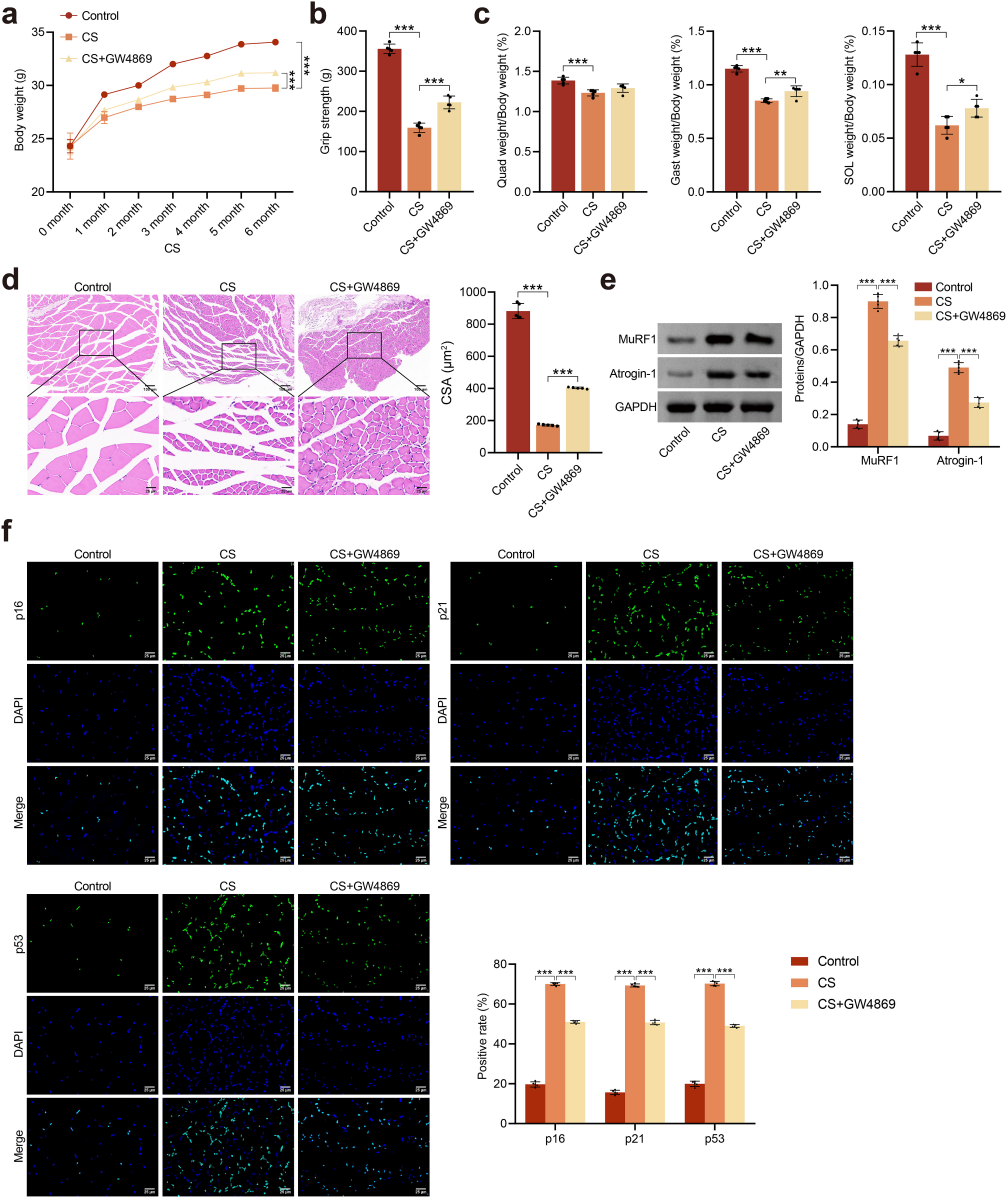
CS exposure induces skeletal muscle senescence and dysfunction in mice via exosomes. (a) Time‐course of body weight changes over 6 months in control and CS‐exposed mice, with or without GW4869 treatment. (b) Grip strength analysis of mice in each group. (c) Relative muscle weights of quadriceps, gastrocnemius and soleus. (d) H&E staining of gastrocnemius sections for CSA measurement (upper: magnification: ×100, scale bar = 100 μm; lower: magnification: ×400, scale bar = 25 μm). (e) Western blot analysis of MuRF1 and Atrogin‐1 in the gastrocnemius muscle. (f) Immunofluorescence staining of p16, p21 and p53 in the gastrocnemius muscle (magnification: ×400, scale bar = 25 μm). *n* = 5; *p < 0.05, **p < 0.01, ***p < 0.001. Statistical significance was determined using two‐way ANOVA (for a) or one‐way ANOVA (for b, c, d, e and f).

To evaluate the effects of CS on skeletal muscle, we established a chronic CS exposure mouse model to simulate COPD‐like pathological manifestations (Figure S1a). CS‐exposed mice showed a progressive decrease in body weight and grip strength (Figure 1a,b), indicating impaired muscle function. The muscle mass of the quadriceps, gastrocnemius and soleus was significantly decreased (Figure 1c), accompanied by reduced fibre CSA (Figure 1d). At the molecular level, CS exposure upregulated the muscle atrophy‐associated ligases MuRF1 and Atrogin‐1 (Figure 1e) and senescence markers p16, p21 and p53 (Figure 1f). Exosome inhibitor GW4869 partially reversed these functional, structural and molecular alterations (Figure 1a–f), suggesting that CS‐induced SMD with cellular senescence is partially mediated by exosomes.

### 3.2 BALF‐Derived Exosomes From CS‐Exposed Mice Induce Skeletal Muscle Senescence and Dysfunction

**FIGURE 2:**
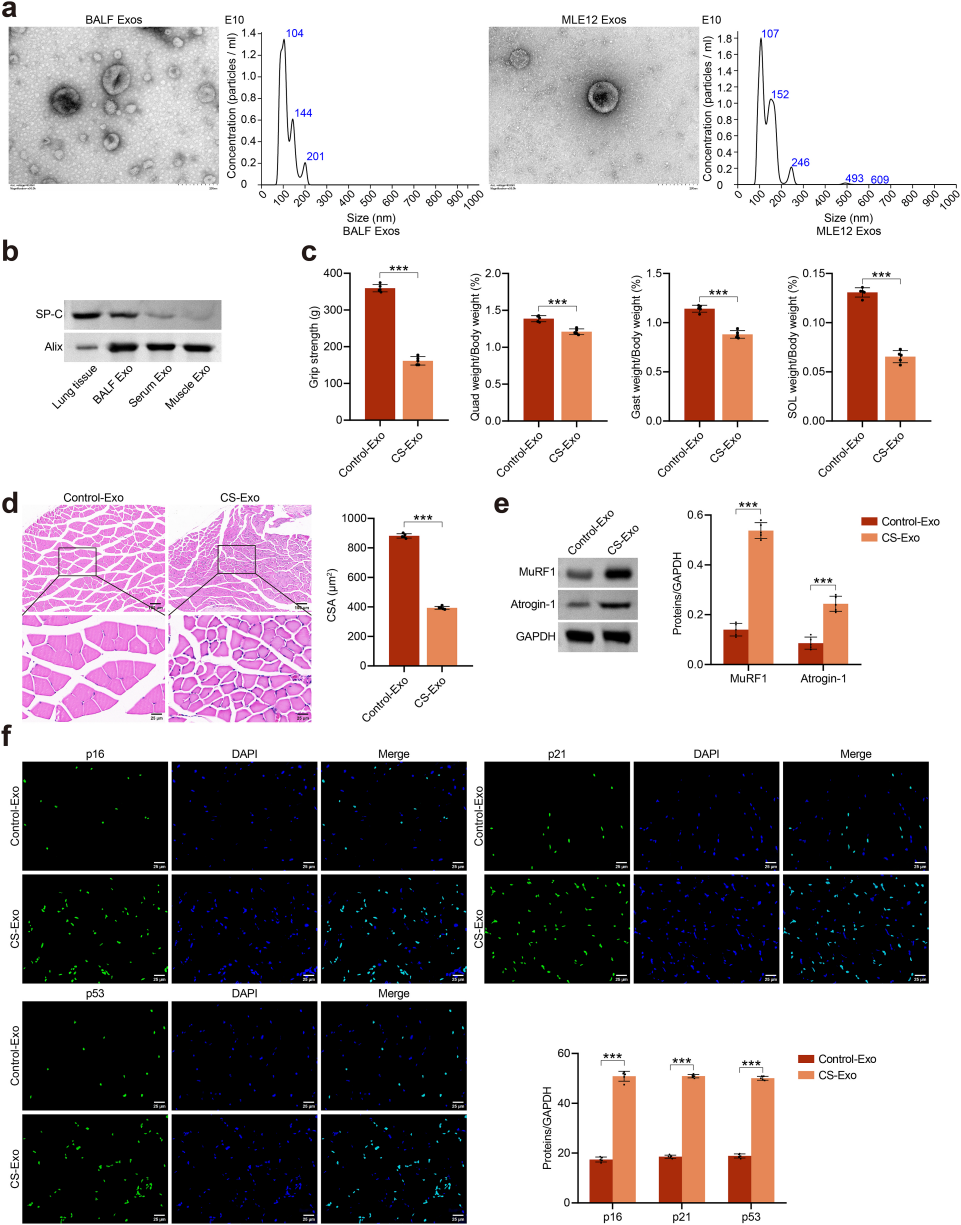
BALF‐derived exosomes from CS‐exposed mice induce skeletal muscle senescence and dysfunction. (a) TEM of exosome morphology and NTA of particle size distribution. (b) Western blot detection of lung‐specific marker SP‐C. (c) Grip strength analysis of mice in each group and relative muscle weights of quadriceps, gastrocnemius and soleus. (d) H&E staining of gastrocnemius sections for CSA measurement (upper: magnification: ×100, scale bar = 100 μm; lower: magnification: ×400, scale bar = 25 μm). (e) Western blot analysis of MuRF1 and Atrogin‐1 in the gastrocnemius muscle. (f) Immunofluorescence staining of p16, p21 and p53 in the gastrocnemius muscle (magnification: ×400, scale bar = 25 μm). *n* = 5; ***p < 0.001. Statistical significance was determined using an unpaired two‐tailed Student's *t*‐test.

To determine whether lung‐derived exosomes contribute to CS‐induced skeletal muscle injury, we isolated BALF‐derived exosomes from control and CS‐exposed mice (Control‐Exo and CS‐Exo). TEM and NTA showed typical cup‐shaped morphology and a particle size of 116.6 ± 31.3 nm (Figure 2a, left panel). They showed enrichment of exosome markers CD63 and TSG101 and absence of cellular marker GM130 (Figure S1c). PKH67 fluorescence tracing further confirmed that BALF‐derived exosomes were efficiently taken up by C2C12 myotubes (Figure S1d). Moreover, the lung‐specific marker SP‐C was detected in lung tissue and BALF‐derived exosomes and was also detectable in circulating (serum) and skeletal muscle‐derived exosomes from CS‐exposed mice (Figure 2b), indicating that lung‐derived exosomes can enter the circulation and reach peripheral tissues, including skeletal muscle. In healthy recipient mice, CS‐Exo reduced grip strength and the mass of quadriceps, gastrocnemius and soleus muscle (Figure 2c), accompanied by decreased gastrocnemius myofiber CSA (Figure 2d). CS‐Exo also upregulated MuRF1, Atrogin‐1, p16, p21 and p53 in gastrocnemius muscle (Figure 2e,f). These findings indicate that CS‐Exo is sufficient to elicit functional and structural abnormalities in skeletal muscle.

### 3.3 CSE‐Exposed Alveolar Epithelial Cell‐Derived Exosomes Induce Senescence and Myogenic Defects in C2C12 Cells

We co‐cultured CS‐Exo with C2C12 myotubes and found that CS‐Exo significantly reduced cell viability and myotube diameter; suppressed MyHC, MyoD and MyoG expression; and increased SA‐β‐gal positivity and p16, p21 and p53 levels (Figure S2a–e), demonstrating that CS‐Exo exerted direct cytotoxic effects on C2C12 cells.

To further model the crosstalk between alveolar epithelial cells and skeletal muscle cells, we established an in vitro co‐culture system in which C2C12 cells were treated with conditioned medium from CSE‐exposed MLE12 cells. Conditioned medium from CSE‐exposed MLE12 cells dose‐dependently reduced C2C12 viability (Figure S3a), myotube diameter (Figure S3b) and MyHC, MyoD and MyoG expression (Figure S3c). It also increased SA‐β‐gal positivity (Figure S3d) and p16, p21 and p53 expression (Figure S3e). However, inhibition of exosome release with GW4869 significantly alleviated all of the aforementioned alterations (Figure S3a–e). The inhibitory effect of GW4869 on exosome release was confirmed by NTA, as indicated by the decreased particle concentration in the culture supernatant (2.32 × 10^10^ vs. 1.66 × 10^11^ particles/mL; Figure S1e).

**FIGURE 3:**
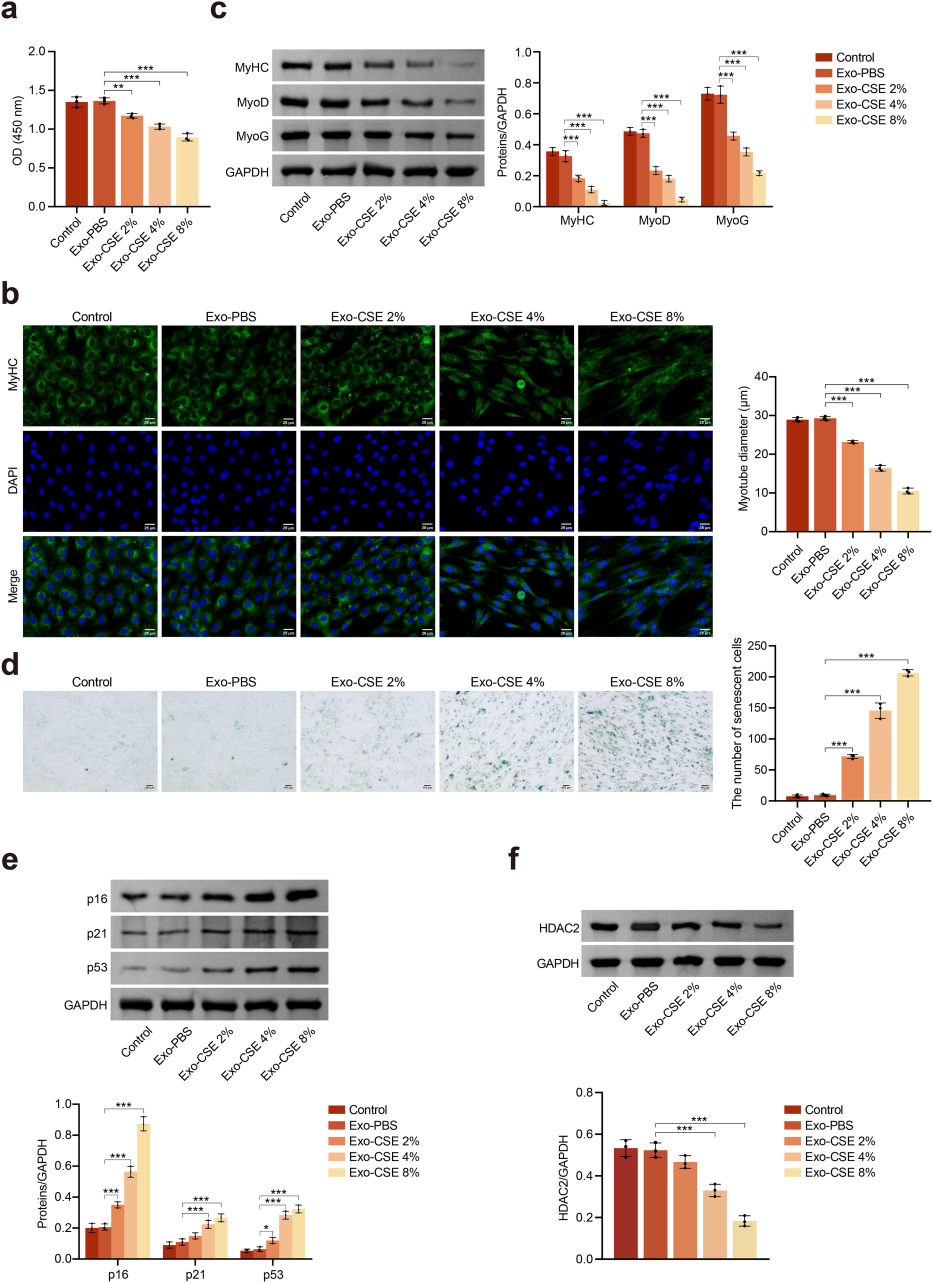
CSE‐exposed alveolar epithelial cell‐derived exosomes induce senescence and myogenic defects in C2C12 cells. (a) Cell viability was measured by CCK8 assay after treatment with exosomes from MLE12 cells exposed to increasing concentrations of CSE. (b) Immunofluorescence staining of MyHC in differentiated myotubes to assess diameter (magnification: ×400, scale bar = 25 μm). (c) Western blot analysis of MyHC, MyoD and MyoG expression. (d) SA‐β‐gal staining of C2C12 cells (magnification: ×100, scale bar = 100 μm). (e) Western blot analysis of p16, p21 and p53 proteins. (f) Western blot analysis of HDAC2 expression after treatment with exosomes from MLE12 cells exposed to CSE. *n* = 3; *p < 0.05, **p < 0.01, ***p < 0.001. Statistical significance was determined using one‐way ANOVA.

Based on these findings, we isolated exosomes directly from MLE12 cells (Exo‐CSE). TEM and NTA revealed typical vesicles with a particle size of 137.9 ± 46.8 nm (Figure 2a, right panel). Western blot confirmed CD63 and TSG101 enrichment and GM130 absence (Figure S1c). PKH67 labeling demonstrated efficient uptake of MLE12‐derived exosomes by C2C12 cells (Figure S1d). Functionally, Exo‐CSE dose‐dependently reduced cell viability (Figure 3a), myotube diameter (Figure 3b) and MyHC, MyoD and MyoG expression (Figure 3c), while increasing SA‐β‐gal positivity (Figure 3d) and p16, p21 and p53 levels (Figure 3e). To explore the downstream signalling mechanism, we examined HDAC2, which is closely associated with COPD and cellular senescence [21]. Exo‐CSE dose‐dependently reduced HDAC2 protein levels in C2C12 cells (Figure 3f). These data demonstrate that Exo‐CSE impairs myogenic differentiation and promotes senescence in C2C12 cells, potentially through HDAC2 signalling.

### 3.4 CSE‐Exposed Alveolar Epithelial Cell‐Derived Exosomes Induce Myogenic Defects in C2C12 Cells via HDAC2 Signalling

To select the CSE concentration, MLE12 cells were exposed to 2%–12% CSE; CSE at 2%–8% did not affect cell viability or apoptosis, whereas 12% CSE exhibited marked cytotoxicity (Figure S4a,b); thus, 8% CSE was used subsequently.

**FIGURE 4:**
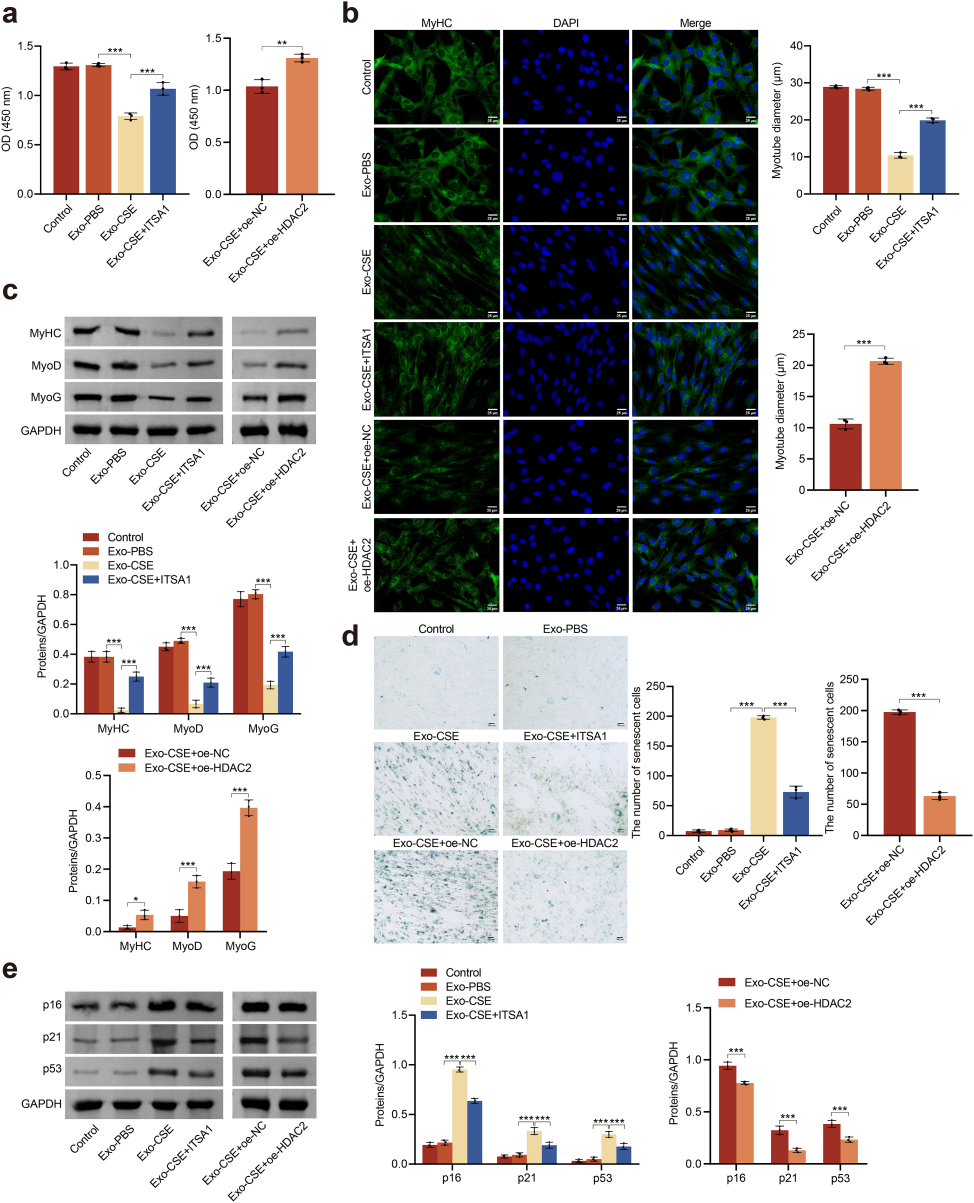
CSE‐exposed alveolar epithelial cell‐derived exosomes induce myogenic defects in C2C12 cells via HDAC2 signalling. (a) Cell viability was measured by the CCK8 assay after exposure to exosomes, with ITSA1 treatment or HDAC2 overexpression. (b) Immunofluorescence staining of MyHC in differentiated myotubes (magnification: ×400, scale bar = 25 μm). (c) Western blot of MyHC, MyoD and MyoG. (d) SA‐β‐gal staining of C2C12 cells. (e) Western blot of p16, p21 and p53. *n* = 3; *p < 0.05, **p < 0.01, ***p < 0.001. Statistical significance was determined using an unpaired two‐tailed Student's *t*‐test for HDAC2 overexpression experiments and one‐way ANOVA for ITSA1 treatment experiments.

Given that HDAC2 was downregulated by Exo‐CSE, we performed rescue experiments in C2C12 cells to determine the functional significance of this reduction. HDAC2 was successfully overexpressed in C2C12 cells (Figure S5a). Both HDAC activator ITSA1 treatment and HDAC2 overexpression significantly reversed the Exo‐CSE‐induced reduction in cell viability (Figure 4a), myotube diameter and MyHC, MyoD and MyoG expression (Figure 4b,c) and reduced senescence, as shown by decreased SA‐β‐gal positivity and p16, p21 and p53 levels (Figure 4d,e). Thus, suppression of HDAC2 signalling is an essential downstream event in the pathogenic effects of Exo‐CSE.

### 3.5 Proteomic Screening Identifies PRELP as a Key Exosomal Cargo That Induces Senescence and Myogenic Defects in C2C12 Cells

**FIGURE 5:**
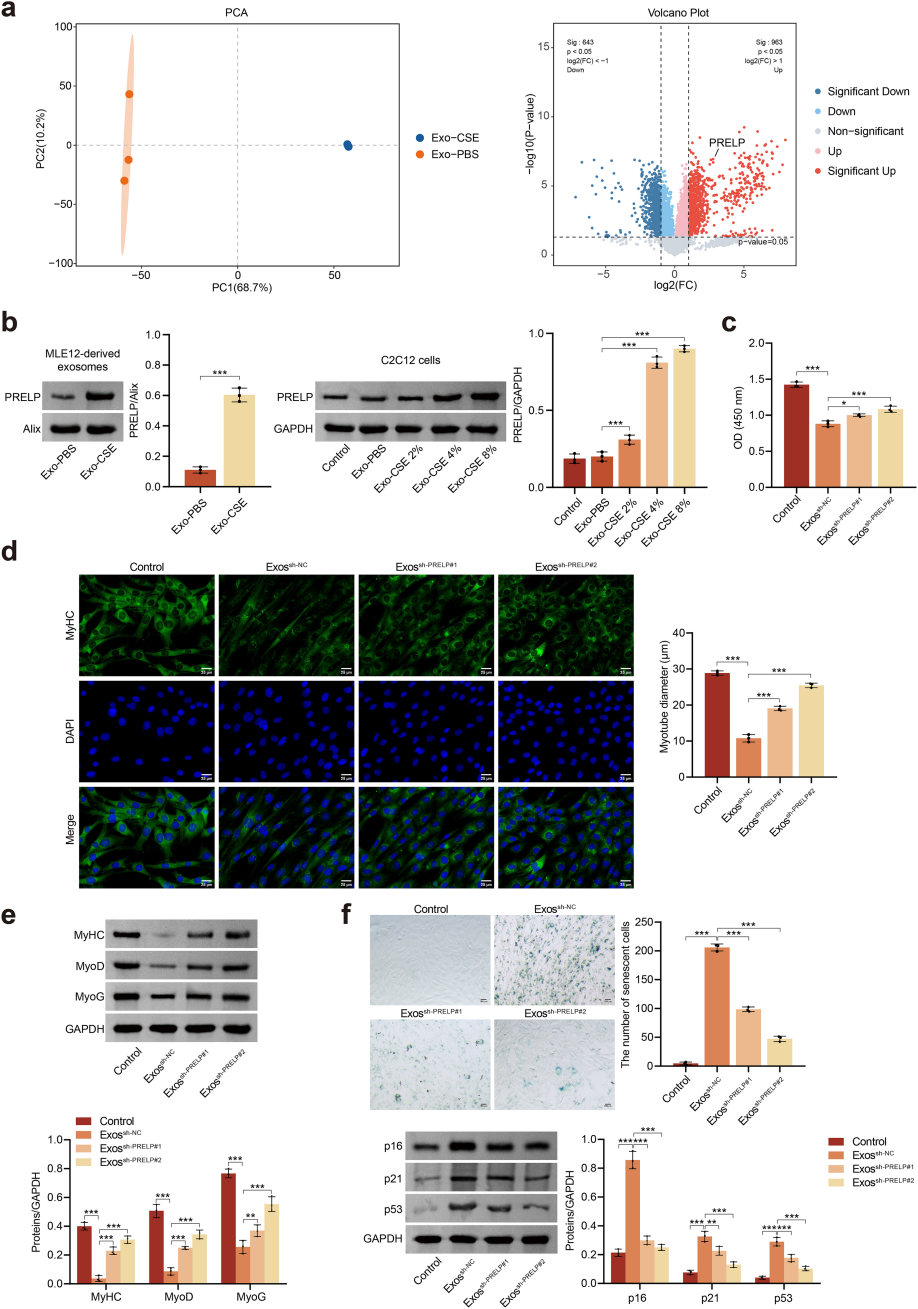
Proteomic screening identifies PRELP as a key exosomal cargo that induces senescence and myogenic defects in C2C12 cells. (a) PCA of proteomic profiles from exosomes derived from PBS‐ and CSE‐treated MLE12 cells and a volcano plot showing differentially expressed proteins. (b) Western blot validation of PRELP expression in exosomes and in C2C12 cells after exosome treatment. (c) CCK8 assay for cell viability. (d) MyHC immunofluorescence staining of differentiated myotubes (magnification: ×400, scale bar = 25 μm). (e) Western blot analysis of MyHC, MyoD and MyoG. (f) SA‐β‐gal staining of C2C12 cells (magnification: ×100, scale bar = 100 μm) and western blot analysis of p16, p21 and p53. Statistical significance was determined using an unpaired two‐tailed Student's *t*‐test (for exosome comparisons in b) or one‐way ANOVA (for cell treatment comparisons in b, and for c, d, e and f).

Alveolar epithelial cells can deliver secretory proteins to other cell types via exosomes [22]. To identify the specific protein cargo in Exo‐CSE responsible for HDAC2 suppression, we performed proteomic profiling of Exo‐PBS and Exo‐CSE. PCA revealed a distinct separation between the Exo‐PBS and Exo‐CSE groups, and volcano plot analysis identified multiple differentially expressed proteins (Figure 5a). Among these, PRELP, a Class II small leucine‐rich proteoglycan (SLRP) reported to increase in aging lung and other organs [23] (Supporting Information Reference S2), was significantly enriched in Exo‐CSE. Western blot confirmed increased PRELP in exosomes from CSE‐treated MLE12 cells and in Exo‐CSE‐treated C2C12 cells in a dose‐dependent manner (Figure 5b). To determine the pathway by which PRELP enters recipient cells, C2C12 cells were pretreated with chlorpromazine, dynasore, simvastatin, or omeprazole before co‐culture with Exo‐CSE. All four endocytosis inhibitors reduced PRELP levels in C2C12 cells (Figure S5b), suggesting that PRELP‐containing exosome uptake involves multiple endocytic mechanisms and membrane fusion.

Then, to evaluate PRELP regulation of HDAC2 and its role in exosome‐mediated muscle injury, we knocked down PRELP in C2C12 cells, which increased HDAC2 expression (Figure S5c). Similarly, exosomes derived from PRELP‐silenced CSE‐exposed MLE12 cells (Exo^sh‐PRELP^) increased HDAC2 levels in recipient C2C12 cells (Figure S5d). Functionally, Exo^sh‐PRELP^ improved C2C12 viability (Figure 5c), increased myotube diameter (Figure 5d) and enhanced MyHC, MyoD and MyoG expression (Figure 5e). It also attenuated senescence, as shown by reduced SA‐β‐gal positivity and decreased p16, p21 and p53 expression (Figure 5f). Together, these findings suggest that exosomal PRELP suppresses HDAC2 expression, thereby promoting senescence and impairing myogenesis in C2C12 cells.

### 3.6 PRELP Suppresses HDAC2 Signalling by Interfering With HSPA5‐HDAC2 Interaction

**FIGURE 6:**
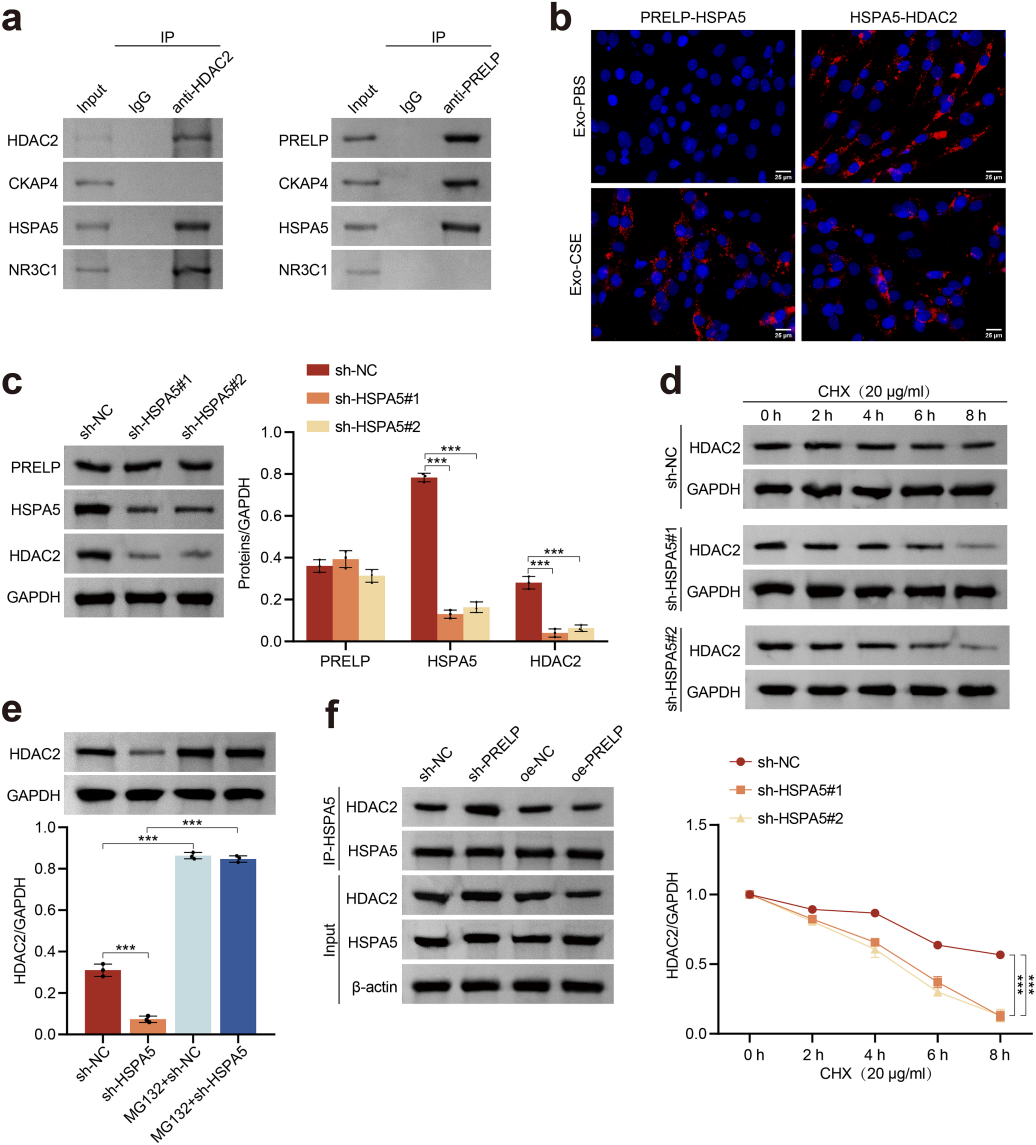
PRELP suppresses HDAC2 signalling by interfering with HSPA5‐HDAC2 interaction. (a) Co‐IP analysis of HDAC2 with CKAP4, HSPA5 and NR3C1 and Co‐IP analysis of PRELP with CKAP4, HSPA5 and NR3C1. (b) PLA analysis of the interaction between PRELP and HSPA5, as well as HSPA5 and HDAC2 (magnification: ×400, scale bar = 25 μm). (c) Western blot analysis of PRELP, HSPA5 and HDAC2 expression after HSPA5 knockdown. (d) CHX chase assay to evaluate HDAC2 protein stability. (e) Western blot analysis of HDAC2 expression levels after treatment with proteasome inhibitor MG132. (f) Co‐IP analysis of HSPA5‐HDAC2 interaction under conditions of PRELP overexpression or knockdown. *n* = 3; ***p < 0.001. Statistical significance was determined using one‐way ANOVA (for c) or two‐way ANOVA (for f).

To elucidate the mechanism by which PRELP regulates HDAC2, we examined potential protein–protein interactions. Co‐IP assays showed no direct interaction between PRELP and HDAC2 (Figure S6a). Based on the BioGRID database, Venn analysis revealed shared candidate interactors, including CKAP4, COL14A1, HSPA5, HSPG2 and NR3C1 (Figure S6b). Because HDAC2 is primarily located in the nucleus and cytoplasm, CKAP4, HSPA5 and NR3C1 were selected for further testing. Co‐IP revealed that HDAC2 interacted with NR3C1 and HSPA5 but not CKAP4, while PRELP interacted with CKAP4 and HSPA5 but not NR3C1 (Figure 6a). PRELP knockdown did not change CKAP4, HSPA5 or NR3C1 levels (Figure S6c), and CKAP4 or NR3C1 silencing did not affect PRELP or HDAC2 (Figure S6d), suggesting that CKAP4 and NR3C1 are unlikely to mediate PRELP regulation of HDAC2. Thus, HSPA5 emerged as the most plausible mediator.

PLA further showed that PRELP‐HSPA5 proximity was detected only in Exo‐CSE‐treated C2C12 cells, whereas HSPA5‐HDAC2 proximity was reduced under Exo‐CSE treatment compared to Exo‐PBS (Figure 6b), suggesting that PRELP disrupts HSPA5 binding to HDAC2.

We next investigated the functional role of HSPA5. HSPA5 silencing reduced HDAC2 protein levels without affecting PRELP (Figure 6c). CHX chase assays showed that HSPA5 silencing accelerated HDAC2 protein degradation (Figure 6d). MG132 treatment increased HDAC2 levels in both control and HSPA5‐silenced cells (Figure 6e), indicating that HSPA5 may protect HDAC2 from ubiquitin‐proteasome‐mediated degradation. Considering that HSPA5 regulates ER stress and client protein stability [24], to exclude ER stress involvement, tunicamycin was used as a positive control. Exo‐CSE treatment did not activate the p‐eIF2α/ATF4/CHOP pathway (Figure S6e), suggesting that the Exo‐CSE‐induced HDAC2 degradation is unlikely to result from ER stress. Co‐IP further confirmed that HSPA5‐HDAC2 interaction was attenuated by PRELP overexpression and strengthened by PRELP knockdown (Figure 6f). These results suggest that PRELP competitively disrupts HSPA5–HDAC2 interaction, thereby abolishing the protective effect of HSPA5 on HDAC2 and accelerating its ubiquitin‐proteasome‐mediated degradation.

### 3.7 PRELP Induces Functional Impairment in C2C12 Cells Largely Through HDAC2 Downregulation

**FIGURE 7:**
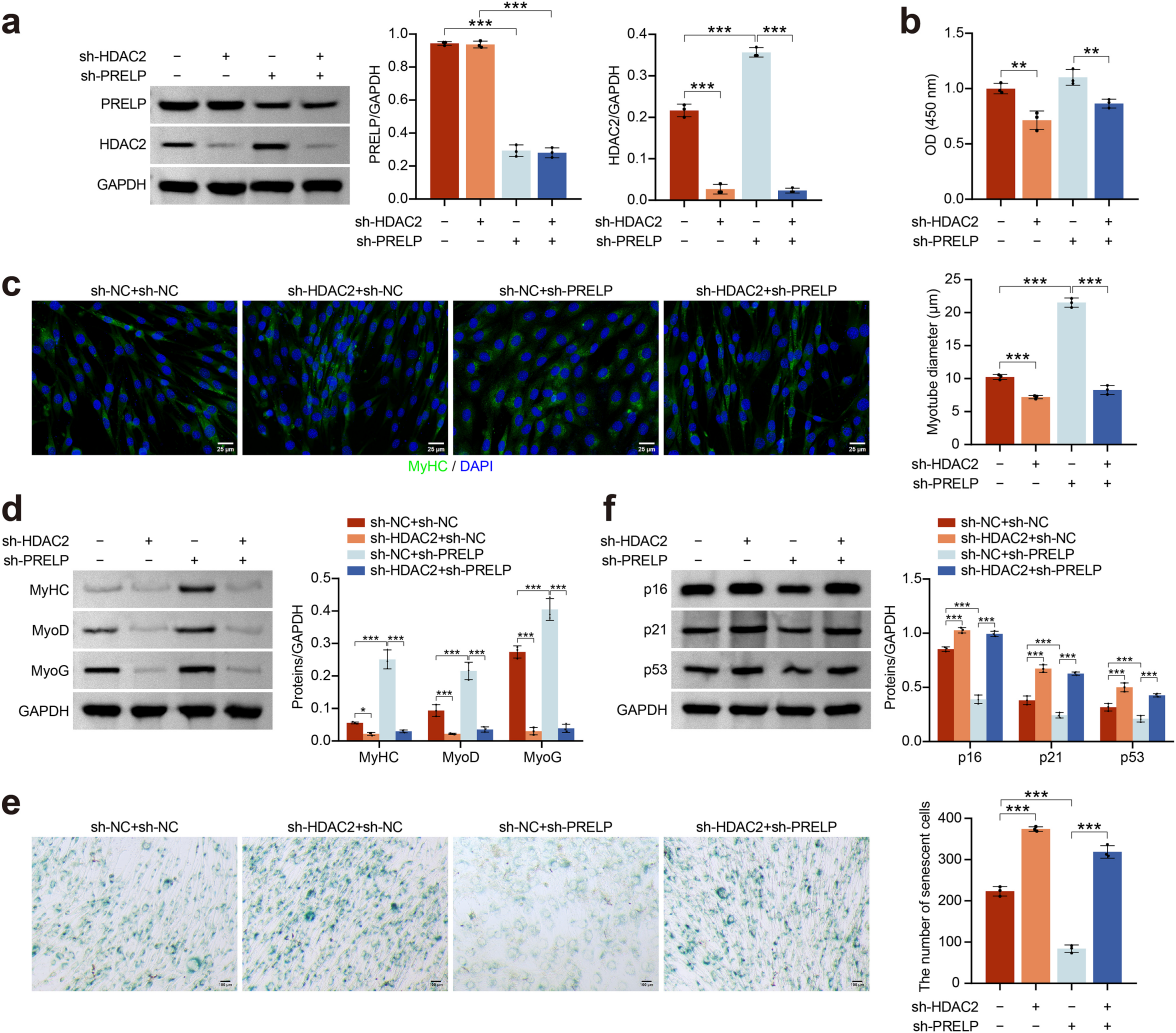
PRELP induces functional impairment in C2C12 cells largely through HDAC2 downregulation. (a) Western blot analysis of PRELP and HDAC2 expression. (b) Cell viability was measured by the CCK8 assay. (c) Immunofluorescence staining of MyHC in differentiated myotubes to assess diameter (magnification: ×400, scale bar = 25 μm). (d) Western blot analysis of MyHC, MyoD and MyoG expression. (e) SA‐β‐gal staining of C2C12 cells (magnification: ×100, scale bar = 100 μm). (f) Western blot analysis of p16, p21 and p53 proteins. *n* = 3; **p < 0.01, ***p < 0.001. Statistical significance was determined using one‐way ANOVA.

Subsequently, we knocked down HDAC2 and/or PRELP in Exo‐CSE‐treated C2C12 cells to test whether PRELP‐mediated injury depends on HDAC2. HDAC2 knockdown reduced HDAC2 expression without affecting PRELP levels, whereas PRELP knockdown decreased PRELP and increased HDAC2 expression (Figure 7a). Functionally, HDAC2 knockdown alone recapitulated the detrimental effects of Exo‐CSE, as evidenced by reduced cell viability (Figure 7b), myotube atrophy (Figure 7c,d) and aggravated cellular senescence (Figure 7e,f), whereas PRELP knockdown exerted protective effects (Figure 7b–f). Notably, concomitant HDAC2 knockdown markedly attenuated the protective effects of PRELP knockdown (Figure 7b–f). These results indicate that PRELP‐induced functional impairment in C2C12 cells is largely dependent on HDAC2 downregulation, identifying HDAC2 as a key downstream effector of PRELP.

### 3.8 PRELP Inhibition Synergizes With the HDAC Activator ITSA1 to Alleviate CS‐Induced SMD in Mice

**FIGURE 8:**
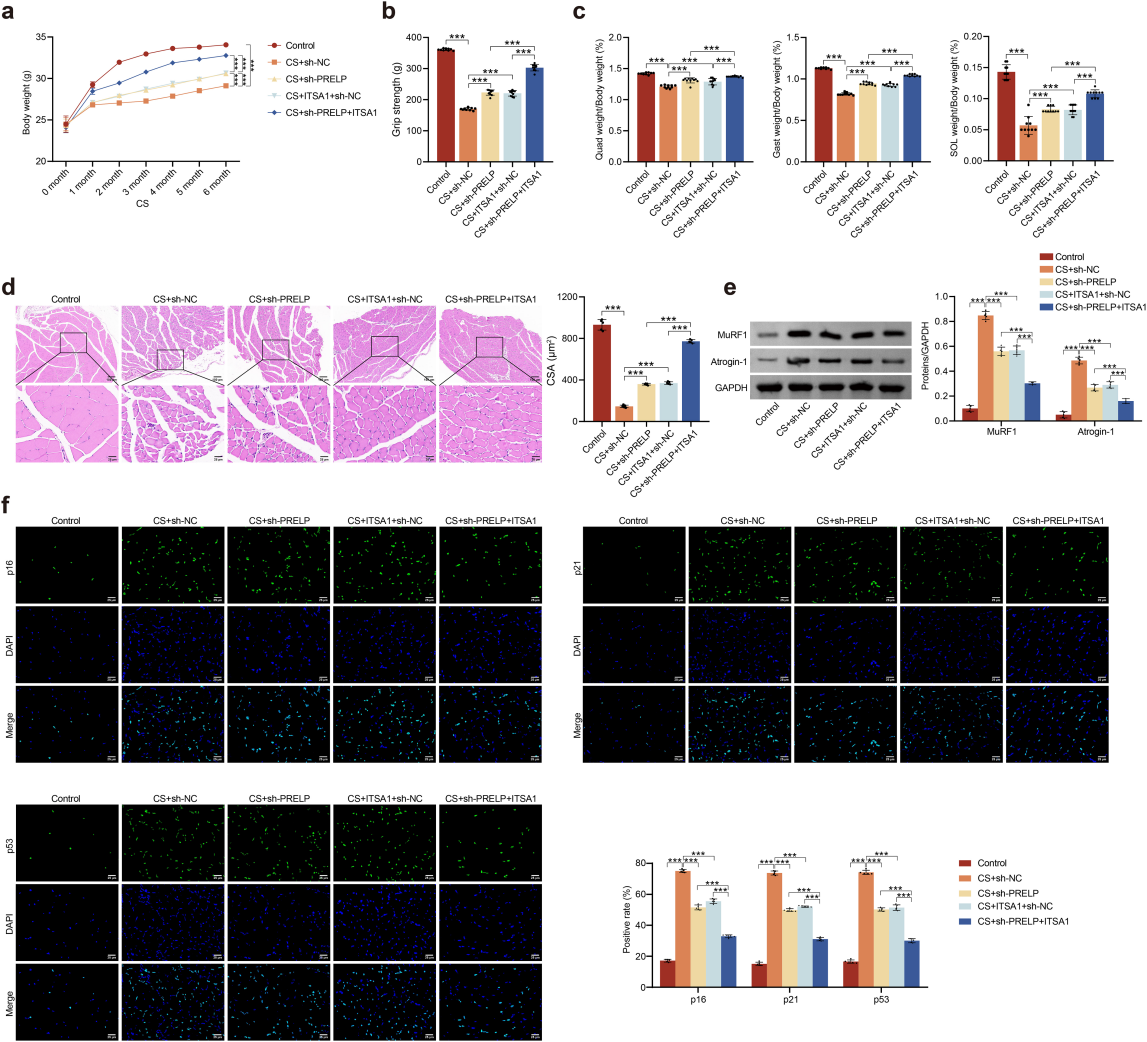
PRELP inhibition synergizes with the HDAC activator ITSA1 to alleviate CS‐induced SMD in mice. (a) Time‐course of body weight changes over 6 months in CS‐exposed mice treated with sh‐PRELP, ITSA1, or combination therapy. (b) Grip strength test of mice in each group. (c) Relative muscle weights of quadriceps, gastrocnemius and soleus. (d) H&E staining of gastrocnemius sections (Upper: magnification: ×100, scale bar = 100 μm; Lower: magnification: ×400, scale bar = 25 μm). (e) Western blot analysis of MuRF1 and Atrogin‐1 in the gastrocnemius muscle. (f) Immunofluorescence staining of p16, p21 and p53 in the gastrocnemius muscle (magnification: ×400, scale bar = 25 μm). *n* = 10 (for a–c), *n* = 5 (for d–f); ***p < 0.001. Statistical significance was determined using two‐way ANOVA (for a) or one‐way ANOVA (for b, c, d, e and f).

To validate the PRELP‐HDAC2 axis in CS‐induced SMD in vivo, mice were intratracheally instilled with AAV‐sh‐PRELP before CS exposure and treated with ITSA1 during CS exposure (Figure S1b). Reduced PRELP levels in lung tissue, BALF‐derived exosomes and skeletal muscle‐derived exosomes confirmed effective in vivo knockdown (Figure S7a–c). CS‐exposed mice exhibited progressive body weight loss, which was partially ameliorated by PRELP knockdown or ITSA1 treatment and most effectively rescued by their combination (Figure 8a). Grip strength and relative weights of quadriceps, gastrocnemius and soleus showed similar partial improvement after either intervention or the greatest recovery after dual treatment (Figure 8b,c). Histological analysis likewise showed increased myofiber CSA, especially with combined treatment (Figure 8d). At the molecular level, sh‐PRELP or ITSA1 reduced CS‐induced MuRF1 and Atrogin‐1 upregulation, with the strongest suppression observed under combined intervention (Figure 8e). Similarly, CS‐induced p16, p21 and p53 expression was alleviated by either treatment and most prominently in the CS + sh‐PRELP+ITSA1 group (Figure 8f). These results suggest that, in COPD‐related SMD, targeting the pathogenic exosomal cargo PRELP while simultaneously activating downstream HDAC2 may represent a promising combination therapeutic strategy.

## 4 Discussion

SMD is a common and debilitating comorbidity of COPD that impairs quality of life and increases mortality risk [25]. Although exosomes are increasingly recognized as mediators of tissue injury in COPD, their involvement in COPD‐related SMD remains unclear [26]. This study identifies a novel mechanism whereby alveolar epithelial cell‐derived exosomal PRELP contributes to CS‐induced SMD by disrupting the HSPA5‐HDAC2 interaction, destabilizing HDAC2 and promoting muscle atrophy and senescence (Figure S7). Through in vivo, in vitro and molecular approaches, we establish a direct mechanistic link between pulmonary injury and muscle dysfunction mediated by exosomal signalling. These findings suggest potential therapeutic strategies targeting exosomal signalling or restoring HDAC2.

In the present study, CS exposure induced SMD in mice in an exosome‐dependent manner, as pharmacological inhibition of exosome release with GW4869 alleviated CS‐induced muscle weakness, atrophy and senescence‐related alterations. Because GW4869 is a systemic nSMase2 inhibitor that ubiquitously suppresses exosome release (Supporting Information Reference S3), the specific exosomal source responsible for CS‐induced SMD required clarification. BALF is an important diagnostic material reflecting alveolar inflammation and injury and contains abundant exosomes (Supporting Information References S4 and S5). BALF‐derived exosomes have also been reported to predict COPD severity and monitor airflow limitation and airway remodelling [27]. In noninfectious lung injury, alveolar epithelial cells are a major source of BALF exosomes [28]. Accordingly, we isolated and characterized BALF‐derived exosomes from mice and demonstrated in vivo and in vitro that they were sufficient to induce SMD‐related phenotypes, supporting the pathogenic role of lung‐derived exosomes. Furthermore, Exo‐CSE impaired myogenic differentiation and enhanced senescence in C2C12 cells, indicating that alveolar epithelial cell‐derived exosomes alone could recapitulate this phenotype. The lung‐specific marker SP‐C was detected in serum and skeletal muscle‐derived exosomes, implying that lung‐derived exosomes may cross the air‐blood barrier and act on skeletal muscle. As polarized cells, alveolar epithelial cells may also release exosomes toward the basolateral or interstitial side, providing another route into the circulation. Feller et al. reported that, in COPD, CS‐exposed alveolar epithelial cell‐derived exosomes carry cytokines through the circulation to adjacent and distant cells, supporting this interpretation [29]. Collectively, these findings suggest a lung‐skeletal muscle crosstalk model in which CS‐exposed alveolar epithelial cells release exosomes into the circulation and contribute to SMD.

HDAC2, an epigenetic regulator with established anti‐inflammatory activity, has been implicated in COPD‐related SMD. Our previous work showed that HDAC2 levels were reduced in skeletal muscle from CS‐exposed mice and contributed to muscle atrophy and senescence through NF‐κB signalling [18]. Here, Exo‐CSE suppressed HDAC2 expression in C2C12 cells, suggesting regulation by lung‐derived exosomal signals during CS exposure. Theophylline has been reported to attenuate skeletal muscle inflammation in a CS‐induced emphysema model by upregulating HDAC2 and decreasing NF‐κB p65 activation [30]. Consistently, HDAC2 overexpression and pharmacological HDAC activation with ITSA1 partially reversed Exo‐CSE‐induced injury in C2C12 cells. Although HDAC2 is best known for repressing inflammatory transcription, evidence suggests that its skeletal muscle role may extend to metabolic homeostasis. Choi et al. showed that PRMT1 deficiency prevents HDAC2 recruitment to the Prmt6 promoter, relieving Prmt6 repression, promoting FOXO3 hyperactivation and inducing excessive autophagy and protein degradation that disrupt muscle homeostasis [31]. Moresi et al. reported that skeletal muscle‐specific double deletion of HDAC1 and HDAC2 causes myopathic degeneration and metabolic abnormalities through impaired autophagic flux [32]. Therefore, beyond regulating myogenic differentiation and senescence, HDAC2 may also contribute to COPD‐related SMD by preserving muscle metabolic homeostasis, which warrants further investigation.

Proteomic analysis identified PRELP as a key cargo of Exo‐CSE, with markedly increased abundance in exosomes from CSE‐exposed MLE12 cells. PRELP is an extracellular matrix protein belonging to the SLRPs family and plays an important role in cell adhesion (Supporting Information References S6 and S7). SLRPs are now recognized not only as structural components but also as signalling regulators that interact with growth factors, cytokines and cell‐surface receptors to modulate cellular processes (Supporting Information Reference S8). Specifically, PRELP should not be viewed merely as an ECM structural protein. Chiavarina et al. reported that fibroblast‐secreted PRELP suppresses hepatocellular carcinoma progression (Supporting Information Reference S9)], whereas Davaapil et al. showed that pericyte‐derived PRELP protects blood–brain barrier integrity (Supporting Information Reference S10), indicating that PRELP may function as an active regulator. Proteomic profiling in an obesity‐associated periodontal remodelling model also identified PRELP as an upregulated protein within the Gene Ontology term “extracellular exosome,” suggesting an association with exosome‐mediated extracellular transport (Supporting Information Reference S11). In our study, all tested endocytosis inhibitors reduced PRELP expression in Exo‐CSE‐treated C2C12 cells, supporting its delivery as an extracellular vesicle cargo. This further suggests that, beyond serving as an extracellular matrix component, PRELP may participate in lung–skeletal muscle crosstalk as an exosomal cargo. Because exosomal cargos often reflect the biological donor cell states, cargos in fluid‐biopsy samples, including blood, have diagnostic potential (Supporting Information Reference S12). Given that PRELP can be detected in serum [33], its potential as a biomarker for COPD‐related SMD appears worthy of further evaluation.

A cross‐tissue analysis of age‐dependent matrisome transcriptome dynamics identified PRELP as significantly upregulated in the heart and lung during aging, implying a role in cellular senescence (Supporting Information Reference S2a). In the present study, Exo‐CSE with PRELP knockdown markedly restored HDAC2 expression in C2C12 cells and alleviated impaired myogenic differentiation and senescence, suggesting that Exo‐CSE may induce COPD‐related SMD through PRELP‐mediated HDAC2 downregulation. Notably, simultaneous HDAC2 knockdown largely abolished the protective effects of PRELP silencing on myogenic differentiation and cellular senescence. This suggests that PRELP functions upstream of HDAC2 by promoting its protein degradation, and thus, PRELP knockdown is insufficient to rescue phenotypic alterations caused by HDAC2 knockdown. Kosuge et al. reported that PRELP suppresses lung cancer cell growth through interactions with IGFI‐R and p75NTR (Supporting Information Reference S13), supporting its signalling‐regulatory capacity and our finding that exosomal PRELP modulates protein homeostasis in recipient cells.

Because no direct interaction was detected between PRELP and HDAC2, their shared interacting proteins were screened, and HSPA5 was ultimately identified as the most plausible bridging molecule. HSPA5, also known as BiP/GRP78, is a key ER chaperone that regulates proteostasis (Supporting Information Reference S14). In skeletal muscle, loss of its co‐chaperone SIL1 disrupts HSPA5 function, leading to ER stress, defective autophagy and premature muscle aging (Supporting Information Reference S15). In lung tissue, HSPA5 exhibits context‐dependent roles: Its overactivation amplifies inflammation and endothelial permeability in acute lung injury (Supporting Information Reference S16), whereas loss of HSPA5 in epithelial progenitors drives ER stress‐induced apoptosis, senescence, and pulmonary fibrosis, especially in aged animals (Supporting Information Reference S17). Our CHX‐chase and MG132‐rescue experiments indicated that HSPA5 maintained HDAC2 protein stability and protected it from proteasome‐dependent degradation. These findings suggest that the muscle‐preserving effect of HSPA5 may depend not only on its expression level but also on its chaperone‐mediated stabilization of key client proteins such as HDAC2. Li et al. recently reported that USP47 enhances HDAC2 stability by promoting its deubiquitination in CS‐induced skeletal muscle atrophy, which further supports the involvement of the ubiquitin‐proteasome pathway in HDAC2 degradation [34]. However, as HSPA5 lacks intrinsic ubiquitin ligase or deubiquitinase activity, we speculate that an unrecognized E3 ubiquitin ligase, such as HRD1 [35] or RLIM [36], may be recruited to facilitate the degradation of HDAC2 within this regulatory cascade. Considering that HSPA5 is classically regarded as an ER protein, how it stabilizes the nuclear protein HDAC2 remains to be clarified. Our PLA data demonstrated HSPA5–HDAC2 interaction signals in the cytoplasm of C2C12 cells. Additionally, several studies have shown that HSPA5 can directly bind to and stabilize other proteins. For example, Gui et al. reported that HSPA5 directly binds to the transcriptional co‐activators YAP/TAZ, which also function in the nucleus, and that this protective interaction suppresses YAP/TAZ ubiquitin‐proteasome degradation [37]. Zhu et al. found that HSPA5 binds to GPX4 and protects it from protein degradation, thereby mediating ferroptosis resistance in pancreatic ductal adenocarcinoma [24], which was later confirmed in colorectal cancer by Wang et al. [38]. Together, these findings suggest that, although HSPA5 is classically regarded as an ER chaperone, it may stabilize HDAC2 through direct interaction in the cytoplasm.

Further mechanistic investigation indicated that the binding and stabilizing effect of HSPA5 on HDAC2 was disrupted by exosomal PRELP, as PRELP interacted with HSPA5, reduced the interaction between HSPA5 and HDAC2 and thereby decreased HDAC2 stability, ultimately promoting its degradation. A recently published review has pointed out that, compared with nucleic acid cargos, the protein cargos of extracellular vesicles remain relatively understudied, highlighting an important gap in current mechanistic understanding [39]. Distinct from the more commonly reported exosomal miRNA‐mediated transcriptional or post‐transcriptional regulation, the present study reveals a mechanism driven by a natural exosomal protein cargo that appears to act primarily by modulating protein stability in recipient cells.

In vivo, treatment of CS‐exposed mice with sh‐PRELP or the HDAC activator ITSA1 alone or in combination was found to alleviate COPD‐related SMD, with the dual intervention showing an apparent synergistic benefit. Enhancement of HDAC2 activity by agents such as theophylline, nortriptyline and macrolides has been regarded as a promising therapeutic strategy for COPD, because it may help reverse glucocorticoid resistance [40]. Previous studies have further shown that theophylline and resveratrol can prevent CS‐induced skeletal muscle atrophy, senescence and inflammation by upregulating HDAC2 expression [30, S1]. The present study further provides an upstream explanation for HDAC2 downregulation in COPD‐related skeletal muscle by showing that lung‐derived exosomal PRELP impairs HDAC2 stability. Therefore, concomitant targeting of the upstream inhibitory factor PRELP may represent an effective adjunctive strategy to enhance HDAC2‐directed therapy for COPD‐related SMD.

Integrating these findings, a model can be proposed in which exosomes act as vehicles that deliver pathogenic cargos such as PRELP, thereby destabilizing HSPA5‐HDAC2 interactions and amplifying SMD. This axis explains how pulmonary stress signals can propagate to distal tissues like skeletal muscle, linking COPD lung injury to muscle atrophy. Nevertheless, our study has limitations. While we demonstrate PRELP‐HSPA5‐HDAC2 interplay, the upstream determinants of exosomal PRELP enrichment under CS remain unclear. Additionally, our work focuses on murine and cell culture models; validation in human cohorts with circulating exosomal profiling is needed. Another limitation is that we did not directly track the accumulation of lung‐derived exosomes in skeletal muscle in vivo. Future studies will employ an in vivo imaging system to directly verify the delivery of lung‐derived exosomes to skeletal muscle.

In conclusion, our work uncovers a novel exosome‐mediated mechanism driving COPD‐related SMD, centred on the PRELP‐HSPA5‐HDAC2 axis. These findings demonstrate how pulmonary injury signals can propagate to skeletal muscle through exosomal cargos, leading to impaired differentiation and accelerated senescence. By revealing this pathway, we highlight the potential of targeting exosomal PRELP or stabilizing HDAC2 as promising therapeutic strategies to alleviate both pulmonary and muscular complications of COPD.

## Funding

This study was supported by the National Natural Science Foundation of China (no. 82400050), the Natural Science Foundation of Hunan Province (no. 2024JJ6270), the Innovation Platform and Talent Plan Project of Hunan Provincial Department of Science and Technology (no. 2023SK4056) and the Young Doctor Foundation of Hunan Provincial People's Hospital (no. BSJJ202215) and Scientific research Project of the Furong Laboratory of the Department of Science and Technology of Hunan Province (no.2023SK2110‐2) .

## Ethics Statement

The study was approved by the Animal Ethics Committee of Hunan Provincial People's Hospital and The First‐Affiliated Hospital of Hunan Normal University under approval number 202442. This study was reported in accordance with the ARRIVE guidelines and performed under related regulations.

## Consent

The authors have nothing to report.

## Conflicts of Interest

The authors declare no conflicts of interest.

## Supporting information




**Figure S1:** Schematic diagram of CS exposure and related interventions in mice and exosomes characteristics. (a) Schematic diagram showing the experimental design and timeline of chronic CS exposure and GW4869 treatment in mice. The initiation of CS exposure was defined as 0 month, and GW4869 treatment was initiated after 3 months of exposure. (b) Schematic diagram showing the experimental design and timeline of AAV‐shPRELP delivery, CS exposure and ITSA1 treatment in mice. CS exposure was defined as 0 month and was initiated 2 weeks after AAV infection. ITSA1 administration started after 4 months of CS exposure. (c) Western blot analysis of exosomal markers CD63 and TSG101 and negative marker GM130. (d) PKH67‐labelled exosomes visualized in C2C12 cells by fluorescence microscopy (magnification: ×400, scale bar = 25 μm). (e) NTA of MLE12‐derived exosomes with or without treatment with GW4869. *n* = 3 (for c and d).
**Figure S2:** CS‐exposed mice BALF‐derived exosomes induce senescence and myogenic defects in C2C12 cells. (a) Cell viability measured by CCK8 assay after treatment with exosomes from the BALF of CS‐exposed mice. (b) Immunofluorescence staining of MyHC in differentiated myotubes to assess diameter (magnification: ×400, scale bar = 25 μm). (c) Western blot analysis of MyHC, MyoD and MyoG expression. (d) SA‐β‐gal staining of C2C12 cells (magnification: ×100, scale bar = 100 μm). (e) Western blot analysis of p16, p21 and p53 proteins. n = 3; ***p < 0.001. Statistical significance was determined using an unpaired two‐tailed Student's t‐test.
**Figure S3:** Conditioned medium from CSE‐exposed epithelial cells induces senescence and myogenic defects in C2C12 cells. (a) Cell viability measured by CCK8 assay after treatment with conditioned medium from PBS‐ or CSE‐exposed MLE12 cells. (b) Immunofluorescence staining of MyHC in differentiated myotubes to assess diameter (magnification: ×400, scale bar = 25 μm). (c) Western blot analysis of MyHC, MyoD and MyoG expression. (d) SA‐β‐gal staining of C2C12 cells (magnification: ×100, scale bar = 100 μm). (e) Western blot analysis of p16, p21 and p53 proteins. n = 3; *p < 0.05, **p < 0.01, ***p < 0.001. Statistical significance was determined using one‐way ANOVA.
**Figure S4:** Screening of CSE intervention concentration. (a) Cell viability measured by CCK8 assay after treatment with increasing concentrations of CSE. (b) Cell apoptosis detected by TUNEL assay after treatment with increasing concentrations of CSE (magnification: ×200, scale bar = 50 μm). ***p < 0.001. Statistical significance was determined using one‐way ANOVA.
**Figure S5:** Validation of HDAC2 overexpression and analysis of PRELP‐HDAC2 regulation. (a) Validation of HDAC2 overexpression in C2C12 cells after oe‐HDAC2 plasmid transfection by western blot. (b) Western blot analysis of PRELP in C2C12 cells after endocytosis inhibitor treatment. (c) Western blot analysis of PRELP and HDAC2 expression after PRELP knockdown. (d) Western blot analysis of PRELP and HDAC2 in C2C12 cells treated with exosomes from PRELP‐silenced MLE12 cells. n = 3; *p < 0.05, **p < 0.01, ***p < 0.001. Statistical significance was determined using an unpaired two‐tailed Student's t‐test (for a) or one‐way ANOVA (for b, c and d).
**Figure S6:** Validation of HDAC2 overexpression and analysis showing that CKAP4 and NR3C1 are not involved in PRELP‐mediated regulation of HDAC2 in C2C12 cells. (a) Co‐IP assay examining direct interaction between PRELP and HDAC2. (b) Venn diagram showing predicted interacting proteins of PRELP and HDAC2 based on the BioGRID database. (c) Western blot analysis of CKAP4, HSPA5 and NR3C1 expression in C2C12 cells transfected with sh‐NC or sh‐PRELP. (d) Western blot analysis of PRELP, CKAP4 and HDAC2 in C2C12 cells transfected with sh‐NC or sh‐CKAP4 and of PRELP, NR3C1 and HDAC2 in C2C12 cells transfected with sh‐NC or sh‐NR3C1. (e) Western blot analysis of ER stress‐related proteins p‐eIF2α, ATF4 and CHOP. n = 3; ***p < 0.001. Statistical significance was determined using one‐way ANOVA.
**Figure S7:** Validation of AAV carrying sh‐PRELP knockdown efficiency. (a) Western blot detection of PRELP expression level in lung tissue. (b) Western blot detection of PRELP expression level in lung‐derived exosomes. (c) Western blot detection of PRELP expression level in skeletal muscle‐derived exosomes. n = 3; **p < 0.01, ***p < 0.001. Statistical significance was determined using an unpaired two‐tailed Student's t‐test.
**Figure S8:** Schematic diagram. PRELP‐enriched exosomes released by CS‐exposed alveolar epithelial cells enter the systemic circulation and are taken up by skeletal muscle cells. Exosomal PRELP disrupts the HSPA5‐HDAC2 interaction and promotes ubiquitin‐mediated HDAC2 degradation, thereby inducing skeletal muscle cell senescence and muscle atrophy and ultimately contributing to skeletal muscle dysfunction.
**Table S1:** The information on the antibody.
**Table S2:** shRNA sequences.

## Data Availability

Data will be made available on request.
