# Supplementary material for: Cigarette Smoke‐Exposed Alveolar Epithelial Cell‐Derived Exosomes Exacerbate Skeletal Muscle Dysfunction Through HDAC2 Signalling
Source: J Cachexia Sarcopenia Muscle. 2026 Jul 19;17(4):e70349. doi: 10.1002/jcsm.70349 (PMC13382079; doi:10.1002/jcsm.70349)
Supplement: Supplementary file 1 — Figure S1: Schematic diagram of CS exposure and related interventions in mice and exosomes characteristics. (a) Schematic diagram showing the experimental design and timeline of chronic CS exposure and GW4869 treatment in mice. The initiation of CS exposure was defined as 0 month, and GW4869 treatment was initiated after 3 months of exposure. (b) Schematic diagram showing the experimental design and timeline of AAV‐shPRELP delivery, CS exposure and ITSA1 treatment in mice. CS exposure was defined as 0 month and was initiated 2 weeks after AAV infection. ITSA1 administration started after 4 months of CS exposure. (c) Western blot analysis of exosomal markers CD63 and TSG101 and negative marker GM130. (d) PKH67‐labelled exosomes visualized in C2C12 cells by fluorescence microscopy (magnification: ×400, scale bar = 25 μm). (e) NTA of MLE12‐derived exosomes with or without treatment with GW4869. n = 3 (for c and d). Figure S2: CS‐exposed mice BALF‐derived exosomes induce senescence and myogenic defects in C2C12 cells. (a) Cell viability measured by CCK8 assay after treatment with exosomes from the BALF of CS‐exposed mice. (b) Immunofluorescence staining of MyHC in differentiated myotubes to assess diameter (magnification: ×400, scale bar = 25 μm). (c) Western blot analysis of MyHC, MyoD and MyoG expression. (d) SA‐β‐gal staining of C2C12 cells (magnification: ×100, scale bar = 100 μm). (e) Western blot analysis of p16, p21 and p53 proteins. n = 3; ***p < 0.001. Statistical significance was determined using an unpaired two‐tailed Student's t‐test. Figure S3: Conditioned medium from CSE‐exposed epithelial cells induces senescence and myogenic defects in C2C12 cells. (a) Cell viability measured by CCK8 assay after treatment with conditioned medium from PBS‐ or CSE‐exposed MLE12 cells. (b) Immunofluorescence staining of MyHC in differentiated myotubes to assess diameter (magnification: ×400, scale bar = 25 μm). (c) Western blot analysis of MyHC, MyoD and MyoG expression. (d [file JCSM-17-e70349-s001.docx]

## Supplementary Materials

### Supplementary Methods

**Animals**

Eight to ten-week-old male C57BL/6J mice were purchased from Hunan Slyke Jingda Experimental Animal Co., Ltd. (Changsha, China) **and** housed under standard conditions (22-25℃, 12-h light/dark cycle) with free access to food and water. **Experiments were initiated after one week of acclimatization.**

Experiment 1: CS exposure and GW4869 treatment. Mice were randomly assigned to Control, CS, and CS + GW4869 **groups (n = 5 per group)**. **Control mice** were **maintained** without CS exposure. Mice in the CS and CS + GW4869 groups were exposed to CS **from five Jinsheng-brand cigarettes (Jiangxi Tobacco Industrial Co., Ltd., Nanchang, China) in a custom-designed chamber (60×50×40 cm) four times daily, five days per week, for six months** [S18, S19]. The start of CS exposure was **defined as** 0 months. After 3 months of CS exposure, mice in the CS + GW4869 group **received intraperitoneal injections of the exosome inhibitor GW4869** (**2.5 mg/kg;** HY-19363, MedChemExpress, Monmouth Junction, NJ, USA) every other day [S20].

Experiment 2: Intervention with bronchoalveolar lavage fluid (BALF)-derived exosomes. Healthy recipient mice were randomly assigned to Control-Exo and the CS-Exo **groups (n = 5 per group)**. **Mice received tail-vein injections of BALF-derived exosomes collected from control or CS-exposed mice, respectively.** Exosomes were administered three times per week at 15 μg per injection in 100 μL for 8 weeks [S21].

Experiment 3: CS exposure combined with PRELP knockdown and ITSA1 treatment. Mice were randomly assigned to Control, CS + sh-NC, CS + sh-PRELP, CS + ITSA1 + sh-NC, and CS + sh-PRELP + ITSA1 **groups (n = 5 per group)**. Two weeks before CS exposure, mice in the sh-PRELP **groups** were intratracheally instilled under anesthesia with adeno-associated virus (AAV) carrying short hairpin RNA (shRNA) targeting PRELP (sh-PRELP)**, whereas the corresponding control groups received sh-NC**. CS exposure was performed as described above. After 4 months of CS exposure, mice in the **ITSA1-treated groups** received intraperitoneal injections of the HDAC activator ITSA1 (0.5 mg/kg; HY-100508, MedChemExpress) three times per week for 8 weeks [S22].

At the end of 6 months, **grip strength was measured** using an electronic force gauge, with three trials per mouse and the average **value** recorded [S23]. **Mice** were **then** euthanized by intraperitoneal injection of an overdose of sodium pentobarbital (150 mg/kg)**.** **Quadriceps**, gastrocnemius, and soleus muscles were dissected and weighed. Muscle mass was normalized to body weight, and gastrocnemius samples were collected for histological and biochemical analyses.

**Cigarette smoke extract (CSE) preparation and treatment**

One unfiltered cigarette was combusted, and the smoke was passed through 4 mL of PBS. The solution was filtered through a 0.22 μm filter**,** adjusted to **pH** 7.00-7.40**, defined as 100% CSE, and** diluted with PBS within 1 h **before** use. **Cells** were treated with CSE for 48 h [S19]. To evaluate the effects of epithelial cell-derived exosomes on myoblasts, C2C12 cells were treated with exosomes isolated from **PBS- or CSE-exposed MLE12 cells, designated Exo-PBS and Exo-CSE, respectively.**

**Hematoxylin and eosin (H&E) staining**

The paraffin-embedded sections of the mouse gastrocnemius were incubated at 60℃ for 2-3 h to soften the paraffin. Sections were then deparaffinized in xylene and rehydrated through a graded ethanol series. Tissue sections were stained with hematoxylin (AWI0001a, Abiowell, Changsha, China) for 1-10 min, rinsed with distilled water, and blued with PBS. The tissue sections were stained with eosin (AWI0029a, Abiowell) for 1-5 min, and then dehydrated in ethanol and cleared in xylene. The sections were mounted with neutral resin and observed under a light microscope.

To assess muscle morphology, the cross-sectional area (CSA) of muscle fibers was measured. Images were captured under 200× magnification, and CSA was quantified using ImageJ software by manually outlining individual muscle fibers. At least 100 myofibers per sample were analyzed to obtain representative averages.

**Exosome isolation and** **characterization**

Exosomes from BALF and serum were isolated as previously reported [S24]. BALF and serum samples were collected and centrifuged to remove cells and debris. Exosomes were then purified using a Plasma/Serum Exosome Isolation and Purification Kit (UR52151, Umibio, Shanghai, China) or a Tissue Exosome Isolation and Purification Kit (UR52161, Umibio) according to the manufacturer’s instructions. Briefly, samples were incubated with the exosome precipitation reagent at 4℃ overnight, centrifuged to obtain the precipitate, resuspended in PBS, and further purified using a size-exclusion chromatography (SEC) column. Purified exosomes were collected and stored at −80℃ until use.

For muscle-derived exosomes, hindlimb muscles, including quadriceps, tibialis anterior, gastrocnemius, and extensor digitorum longus, were dissected, washed, and cut into small pieces. Tissues were cultured in medium containing exosome-depleted FBS at 37℃ with 5% CO2 for 48-72 h. The conditioned medium was filtered through a 70 μm nylon mesh, centrifuged to remove debris, and passed through a 0.22 μm filter. The filtrate was concentrated using centrifugal filter units (Amicon Ultra-15, #UFC910096, Millipore, Billerica, MA, USA), and exosomes were subsequently purified following the same procedure used for serum samples.

For exosomes derived from MLE12 cells, culture supernatants were collected and sequentially centrifuged to remove floating cells and cell debris, followed by filtration through a 0.22 μm membrane. The supernatant was then concentrated using ultrafiltration tubes and ultracentrifuged at 100,000 ×g for 2 h. The resulting pellet was collected as the exosome fraction and resuspended in PBS for subsequent analyses.

Exosome morphology was examined by transmission electron microscopy (TEM; JEM-1400FLASH, JEOL, Tokyo, Japan). Exosome size distribution and concentration were analyzed by nanoparticle tracking analysis (NTA) using NanoSight (ZetaView_Particle Metrix, DKSH, Shanghai, China). Exosome markers CD63 and TSG101, along with GM130, were detected by western blot. All procedures were performed as previously described [S25].

**Exosome uptake assay**

Exosomes were labeled by adding 50 μL of PKH67 working solution (100 μM; MINI67, Sigma, St. Louis, MO, USA) directly to the exosome suspension and incubating in the dark for 30 min. The labeling was terminated by adding an equal volume of 1% BSA solution. The mixture was then diluted with 1 mL of complete medium and added to the recipient cells. After 6 h of incubation at 37℃, cells were washed three times with PBS, fixed with 4% paraformaldehyde, and stained with Hoechst 33342. After an additional three PBS washes, exosome uptake was visualized using a fluorescence microscope.

**Cell culture and treatment**

The mouse C2C12 myoblasts (AW-CNM118, Abiowell) were maintained in DMEM supplemented with 10% fetal bovine serum (FBS) and 1% penicillin/streptomycin. To induce differentiation, cells were grown to 70-80% confluence and then incubated in DMEM containing 2% horse serum for 7 days, with media changes every other day [S26]. The mouse alveolar epithelial cell line MLE12 (AW-CNM486, Abiowell) was cultured in DMEM/F12 supplemented with 10% FBS and 1% penicillin/streptomycin. Both C2C12 and MLE12 cells were maintained in a humidified incubator at 37℃ with 5% CO2.

Experiment 1: Treatment of C2C12 cells with conditioned medium from CSE-exposed MLE12 cells. C2C12 cells were divided into five groups: Control, CSE 2%, CSE 4%, CSE 8%, and CSE 8% + GW4869. Conditioned medium collected from MLE12 cells treated with different concentrations of CSE (2%, 4%, or 8%) was used to treat C2C12 cells for 24 h. In the CSE 8% + GW4869 group, MLE12 cells were pretreated with the exosome inhibitor GW4869 (20 μM) for 12 h before exposure to 8% CSE, and the conditioned medium was then collected for C2C12 treatment.

Experiment 2: Treatment of C2C12 cells with exosomes derived from CSE-exposed MLE12 cells (Exo-CSE) or BALF from CS-exposed mice (CS-Exo). C2C12 cells were divided into seven groups: Control, Exo-PBS, Exo-CSE 2%, Exo-CSE 4%, Exo-CSE 8%, Control-Exo, and CS-Exo. For the first five groups, C2C12 cells were treated for 24 h with exosomes isolated from the conditioned medium of MLE12 cells exposed to PBS or different concentrations of CSE (2%, 4%, or 8%). For the last two groups, C2C12 cells were treated for 24 h with exosomes isolated from the BALF of control mice or CS-exposed mice, designated Control-Exo and CS-Exo, respectively.

Experiment 3: Exposure of MLE12 cells to different concentrations of CSE. MLE12 cells were divided into five groups: Control, CSE 2%, CSE 4%, CSE 8%, and CSE 12%. Cells were exposed to the indicated concentrations of CSE for 48 h to determine the appropriate concentration for subsequent experiments.

Experiment 4: Treatment of C2C12 cells with Exo-CSE in the presence or absence of the HDAC activator ITSA1. C2C12 cells were divided into four groups: Control, Exo-PBS, Exo-CSE, and Exo-CSE + ITSA1. In the Exo-CSE + ITSA1 group, C2C12 cells were co-cultured with exosomes derived from MLE12 cells exposed to 8% CSE and simultaneously treated with HDAC activator ITSA1 (100 μM) for 24 h.

Experiment 5: Treatment of C2C12 cells with endocytosis inhibitors. C2C12 cells were treated with various endocytosis inhibitors for 30 min at 37℃ before co-culture with exosomes derived from MLE12 cells treated with 8% CSE. The inhibitors included chlorpromazine (10 μM**; clathrin-mediated endocytosis inhibitor**), dynasore (20 μM**; actin- and caveolin-mediated endocytosis inhibitor**), simvastatin (1.5 μM**; lipid raft disruptor**), and omeprazole (10 μM**; membrane fusion inhibitor**).

Experiment 6: Tunicamycin-induced endoplasmic reticulum stress. C2C12 cells were divided into four groups: Control, Exo-PBS, Exo-CSE, and tunicamycin. C2C12 cells in the Exo-PBS and Exo-CSE groups were treated with the corresponding exosomes, whereas cells in the tunicamycin group were treated with 10 μg/mL tunicamycin for 1 h as a positive control for endoplasmic reticulum stress.

**Cell transfection**

MLE12 cells or C2C12 cells were plated at a density of 1×10^5^ cells per well in 6-well plates and incubated at 37℃ for 24 h. After this, the cells were transfected with plasmid constructs including control (sh-NC and oe-NC), knockdown (sh-PRELP, sh-CKAP4, sh-NR3C1, sh-HSPA5, sh-HDAC2), and overexpression (oe-PRELP, oe-HDAC2) constructs, with the following vector details: sh-NC (HG-shNC, HonorGene, Changsha, China), sh-PRELP (HG-MO054077-sh, HonorGene), sh-CKAP4 (HG-MO175451-sh, HonorGene), sh-NR3C1 (HG-MO008173-sh, HonorGene), sh-HSPA5 (HG-MO022310-sh, HonorGene), sh-HDAC2 (HG-MO008229-sh, HonorGene), oe-PRELP (HG-HO054077, HonorGene) and oe-HDAC2 (HG-MO008229, HonorGene). The shRNA sequences are shown in **Supplementary Table S2**. Transfection was performed using Lipofectamine 2000 (11668-019, Invitrogen, Carlsbad, CA, USA) according to the manufacturer’s instructions, and transfection efficiency was evaluated 48 h post-transfection.

**Western blot**

Proteins from mouse gastrocnemius muscle and C2C12 cells were extracted using RIPA buffer (P0013B, Beyotime, Shanghai, China), and protein concentrations were determined using the BCA assay (AWB0104, Abiowell). Equal amounts of protein were separated by SDS-PAGE, transferred to nitrocellulose membranes, and blocked with 5% skim milk in PBST. Membranes were incubated overnight at 4℃ with primary antibodies, washed with PBST, and then incubated with HRP-conjugated secondary antibodies. Protein signals were detected using ECL (K-12045-D50, Advansta, San Jose, CA, USA) and captured with a chemiluminescence imaging system (ChemiScope6100, Clinx Science, Shanghai, China). Antibody details are listed in **Supplementary Table S1**.

**Immunofluorescence staining**

C2C12 cells were fixed with 4% paraformaldehyde for 30 min, permeabilized with 0.3% Triton X-100, and blocked with 5% BSA at 37℃ for 1 h. After washing, cells were incubated overnight at 4℃ with a primary antibody against MyHC (1:50; AWA58884, Abiowell), p16 (1:200; ab211542, Abcam), p21 (1:200; ab188224, Abcam), or p53 (1:200; ab26, Abcam), followed by a 1 h incubation at 37℃ with a fluorescent secondary antibody, including goat anti-rabbit IgG (H+L) (AWS0005a, Abiowell) and goat anti-mouse IgG (H+L) (AWS0003, Abiowell) conjugated to Alexa Fluor dyes. After additional PBS washes, coverslips were mounted with anti-fading glycerol and stored in the dark. Fluorescence images were captured using a fluorescence microscope, and myotube diameter was measured.

**Cell viability assay using cell counting kit-8 (CCK-8)**

Cells were trypsinized, counted, and seeded into 24-well plates at 1×10^4^ cells/well. After cell attachment and treatment, the medium was replaced with 300 μL fresh medium containing 10% CCK-8 solution (NU679, Dojindo, Kumamoto, Japan). After 4 h of incubation at 37℃ in 5% CO_2_, absorbance at 450 nm was measured using a microplate reader (MB-530, HEALES, Shenzhen, China). The average OD values from triplicate wells were used to assess cell viability.

**Senescence-associated β-galactosidase (SA-β-gal) staining**

Cells were washed once with PBS, then fixed with 1 mL SA-β-gal fixation solution (C0602, Beyotime) at room temperature for 15 min. After removing the fixative, cells were washed three times with PBS for 3 min each. One milliliter of staining working solution was added per well, prepared by mixing 10 μL solution A, 10 μL solution B, 930 μL solution C, and 50 μL X-Gal solution. Cells were incubated overnight at 37℃ in a non-CO_2_ environment with the plates sealed using parafilm. The next day, SA-β-gal-positive cells were observed and imaged under a light microscope.

**Terminal deoxynucleotidyl transferase dUTP nick end labeling (TUNEL) assay**

MLE12 cells were fixed with 4% paraformaldehyde for 30 min, permeabilized with 0.3% Triton X-100 at 37℃ for 30 min, and digested with Proteinase K working solution for 20 min at 37℃. After equilibration, cells were incubated with TdT reaction buffer containing FITC-12-dUTP labeling mix and recombinant TdT enzyme at 37℃ for 60 min in the dark. Negative control samples were processed in parallel without the TdT enzyme. After PBS washing, nuclei were stained with DAPI for 10 min, and coverslips were mounted with glycerol buffer. Fluorescence images were captured under a fluorescence microscope. The above procedures were performed according to the manufacturer's instructions for the TUNEL Apoptosis Detection Kit (FITC) (40306ES50, YEASEN, Shanghai, China).

**Proteomic and bioinformatics analysis**

Exosomal proteins from control and CSE-exposed alveolar epithelial cells were extracted and enzymatically digested into peptides. The resulting samples were analyzed using a timsTOF HT mass spectrometer (Bruker, Fremont, CA, USA) operated in data-independent acquisition (DIA) mode. The raw spectra were processed through directDIA for peptide identification and quantification, followed by normalization and filtering based on reproducibility across biological replicates. Proteins identified with at least two unique peptides and a false discovery rate (FDR) below 1% were retained for quantitative analysis. The processed dataset was subsequently subjected to proteomic bioinformatics analysis to identify key secreted proteins. Principal component analysis (PCA) and a volcano plot were used to visualize the differentially expressed proteins. Potential interacting molecules of PRELP and HDAC2 were obtained from the BioGRID database (https://thebiogrid.org/), and a Venn diagram was generated to visualize the overlap of their interactions.

**Proximity ligation assay (PLA)**

PLA was performed with Duolink In situ Detection Reagents Red (DUO92008, Sigma) following the manufacturer’s instructions. C2C12 cells treated with Exo-PBS or Exo-CSE were fixed with 4% paraformaldehyde, permeabilized with 0.2% Triton X-100, and blocked with Duolink blocking solution. Cells were then incubated overnight at 4℃ with primary antibodies against PRELP (1:50; ab229719, Abcam), HSPA5 (1:50; 11587-1-AP, Proteintech), or HDAC2 (1:50; 12922-3-AP, Proteintech). After washing, slides were incubated with Duolink PLA PLUS and MINUS probes, followed by ligation and rolling-circle amplification according to the manufacturer’s protocol. Nuclei were counterstained with DAPI, and fluorescence signals were visualized under a fluorescence microscope.

**Cycloheximide (CHX) chase assay and MG132 treatment**

C2C12 cells were transfected with sh-NC or sh-HSPA5 and maintained in complete medium until actively proliferating. To inhibit de novo protein synthesis, cycloheximide (CHX) was added to the culture medium at a final concentration of 100 μg/mL. Cells were harvested at 0, 2, 4, 6, and 8 h after CHX treatment, washed with cold PBS, and lysed in RIPA lysis buffer containing proteasome inhibitors to obtain total protein extracts. HDAC2 protein expression was analyzed by Western blot to assess the impact of HSPA5 knockdown on its stability.

To assess the effect of proteasome inhibitor MG132 on HDAC2 stability under HSPA5 knockdown, C2C12 cells transfected with sh-NC or sh-HSPA5 were treated with 10 μM MG132 (Y16782, Biolab, China) for 3 h, and corresponding controls received an equal volume of DMSO. After treatment, cells were collected and lysed to obtain total protein. Western blot was performed to detect HDAC2 protein levels.

**Co-immunoprecipitation (Co-IP)**

Cells were lysed in 300 μL IP lysis buffer, sonicated for 1.5 min, incubated on ice for 30 min, and centrifuged at 12,000 rpm for 15 min at 4℃. The supernatants were collected and incubated overnight at 4℃ with HDAC2, PRELP, or HSPA5 antibodies, or control IgG (B900610, Proteintech). Protein A/G agarose beads were pre-washed and added to the lysates for 2 h at 4℃. After incubation, the beads were washed four times with lysis buffer, and the final pellet was collected. Co-precipitated proteins were analyzed by western blot.

### Supplementary Figures


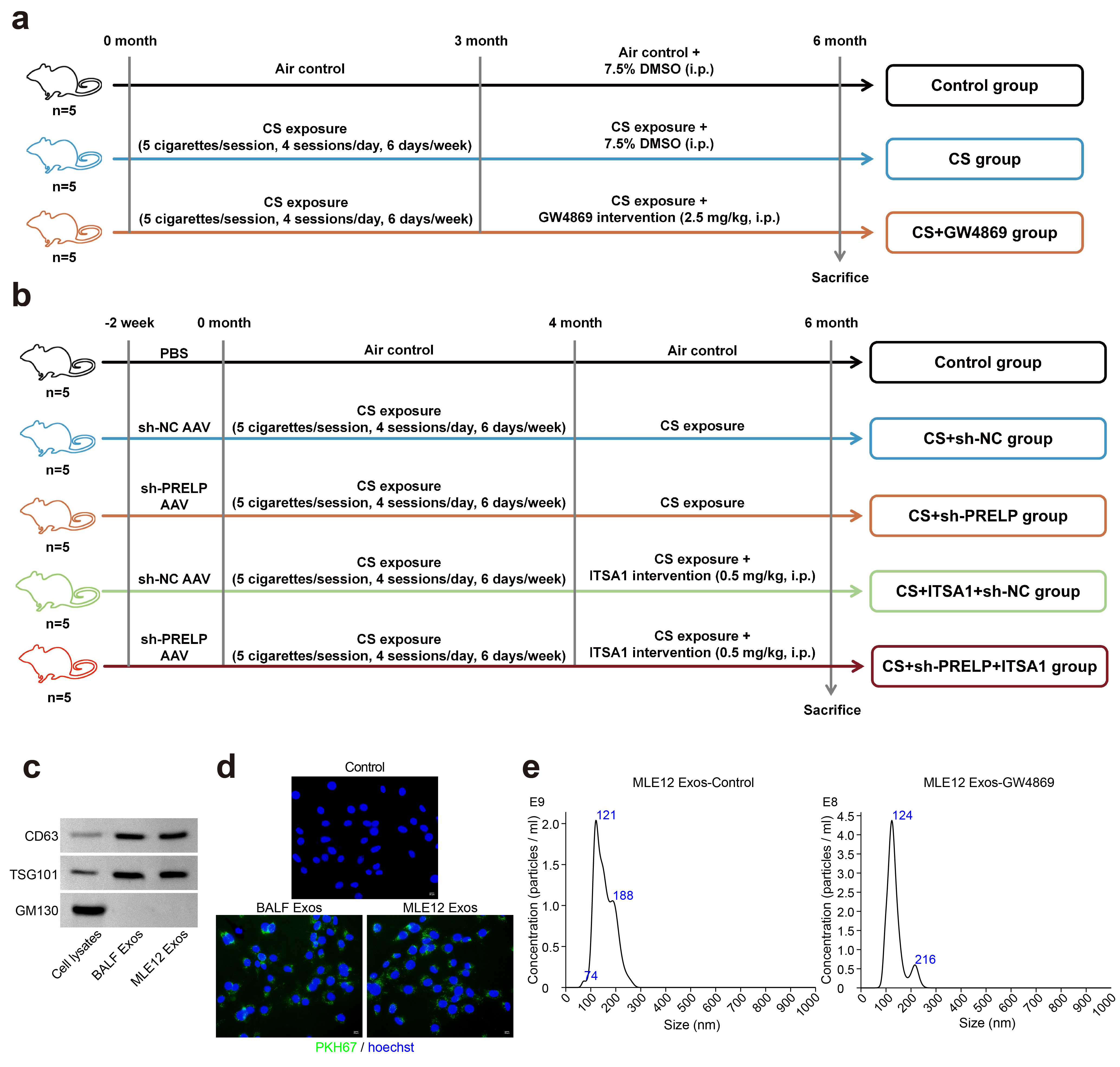


**Supplementary Figure S1. Schematic diagram of CS exposure and related interventions in mice and exosomes characteristics.** (a) Schematic diagram showing the experimental design and timeline of chronic CS exposure and GW4869 treatment in mice. The initiation of CS exposure was defined as 0 month, and GW4869 treatment was initiated after 3 months of exposure. (b) Schematic diagram showing the experimental design and timeline of AAV-shPRELP delivery, CS exposure, and ITSA1 treatment in mice. CS exposure was defined as 0 month and was initiated 2 weeks after AAV infection. ITSA1 administration started after 4 months of CS exposure. **(c) Western blot analysis of exosomal markers CD63 and TSG101 and negative marker GM130. (d) PKH67-labeled exosomes visualized in C2C12 cells by fluorescence microscopy (magnification: ×400, scale bar = 25 μm). (e) NTA of MLE12-derived exosomes with or without treatment with GW4869. n = 3 (for c and d).**


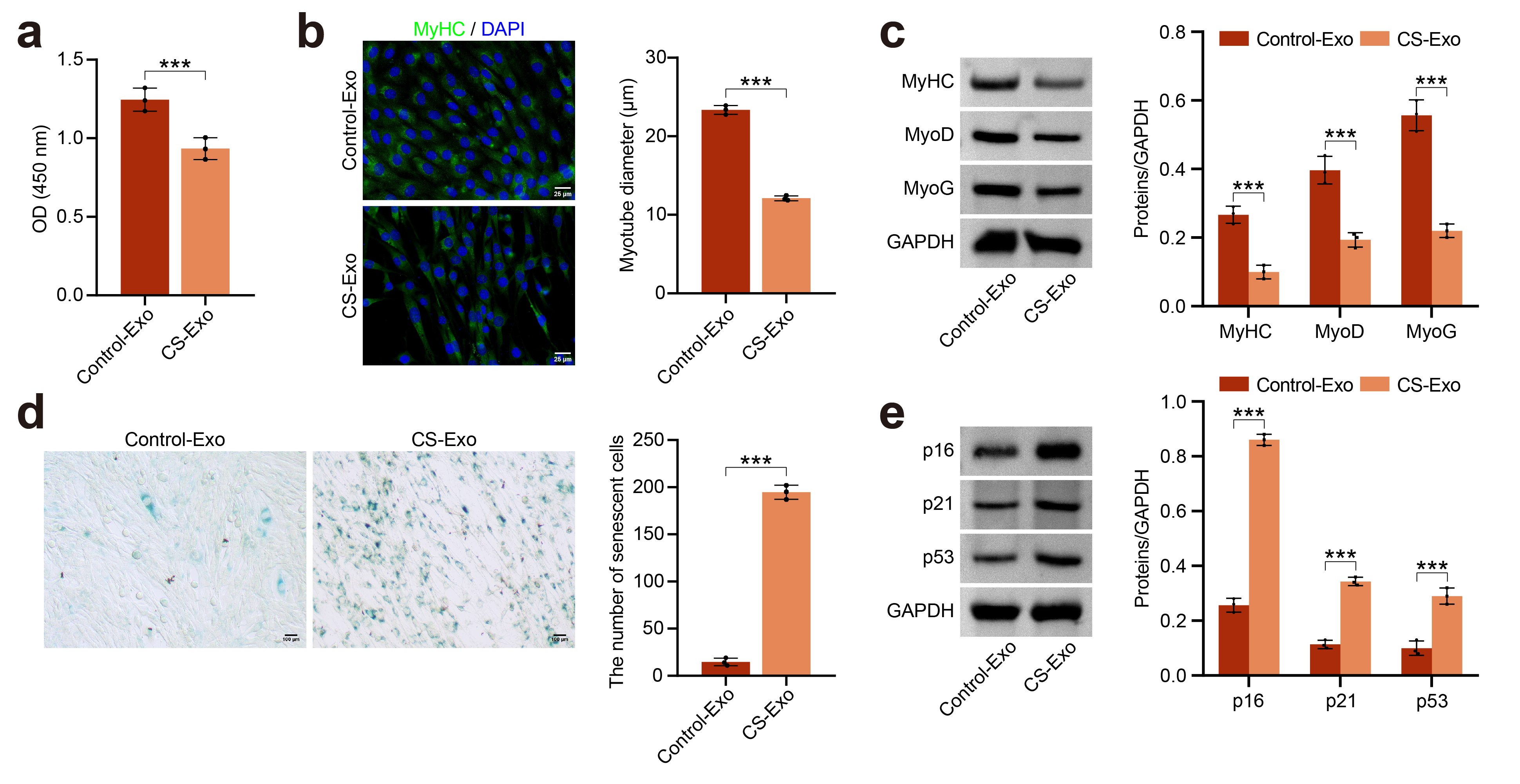


**Supplementary Figure S2. CS-exposed mice BALF-derived exosomes induce senescence and myogenic defects in C2C12 cells.** (a) Cell viability measured by CCK8 assay after treatment with exosomes from the BALF of CS-exposed mice. (b) Immunofluorescence staining of MyHC in differentiated myotubes to assess diameter (magnification: ×400, scale bar = 25 μm). (c) Western blot analysis of MyHC, MyoD, and MyoG expression. (d) SA-β-gal staining of C2C12 cells (magnification: ×100, scale bar = 100 μm). (e) Western blot analysis of p16, p21, and p53 proteins. n = 3; ***p< 0.001. Statistical significance was determined using an unpaired two-tailed Student's *t*-test.


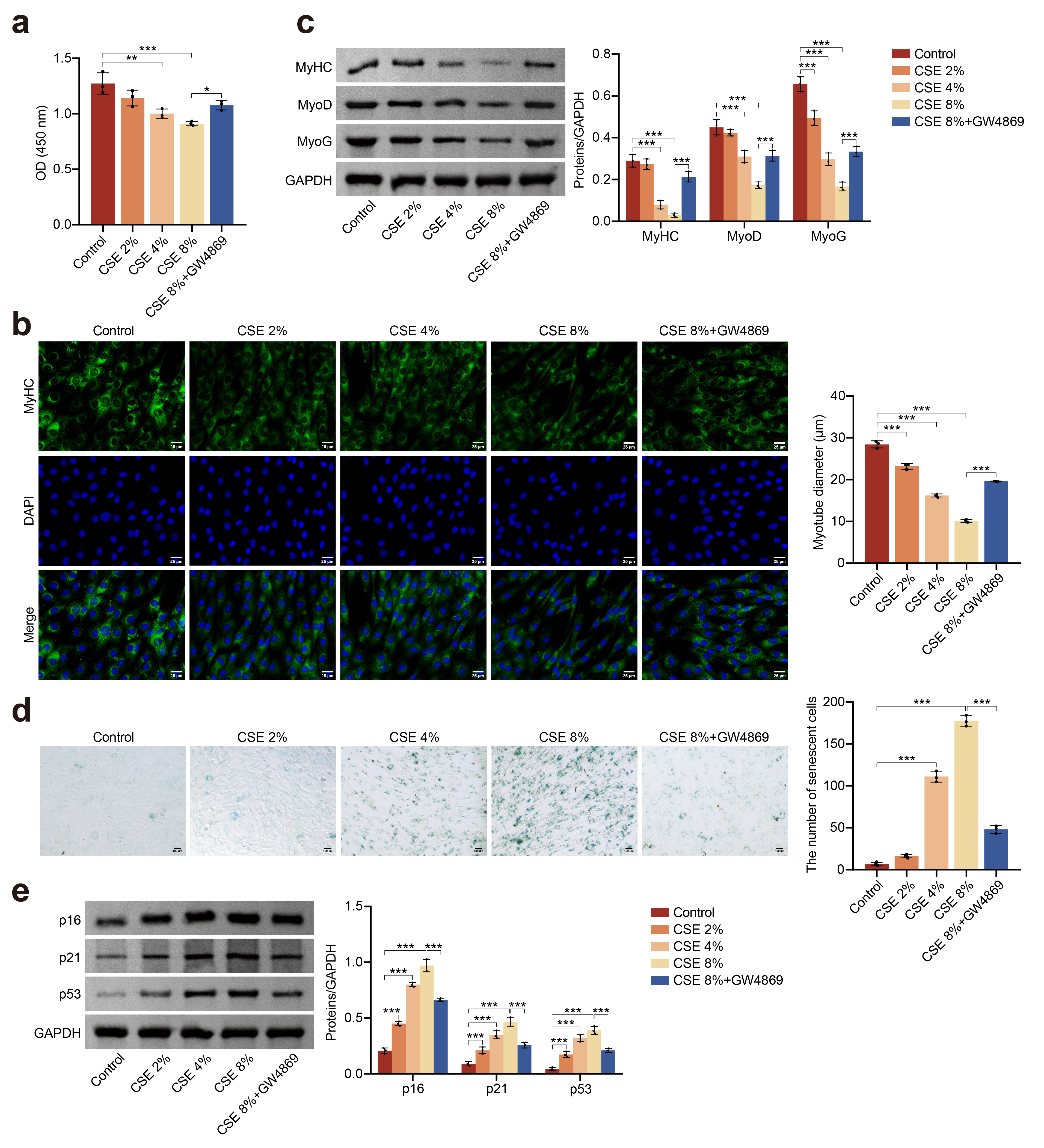


**Supplementary Figure S3. Conditioned medium from CSE-exposed epithelial cells induces senescence and myogenic defects in C2C12 cells.** (**a**) Cell viability measured by CCK8 assay after treatment with conditioned medium from PBS- or CSE-exposed MLE12 cells. (**b**) Immunofluorescence staining of MyHC in differentiated myotubes to assess diameter (magnification: ×400, scale bar = 25 μm). (**c**) Western blot analysis of MyHC, MyoD, and MyoG expression. (**d**) SA-β-gal staining of C2C12 cells (magnification: ×100, scale bar = 100 μm). (**e**) Western blot analysis of p16, p21, and p53 proteins. n = 3; *p<0.05, **p<0.01, ***p< 0.001. Statistical significance was determined using one-way ANOVA.


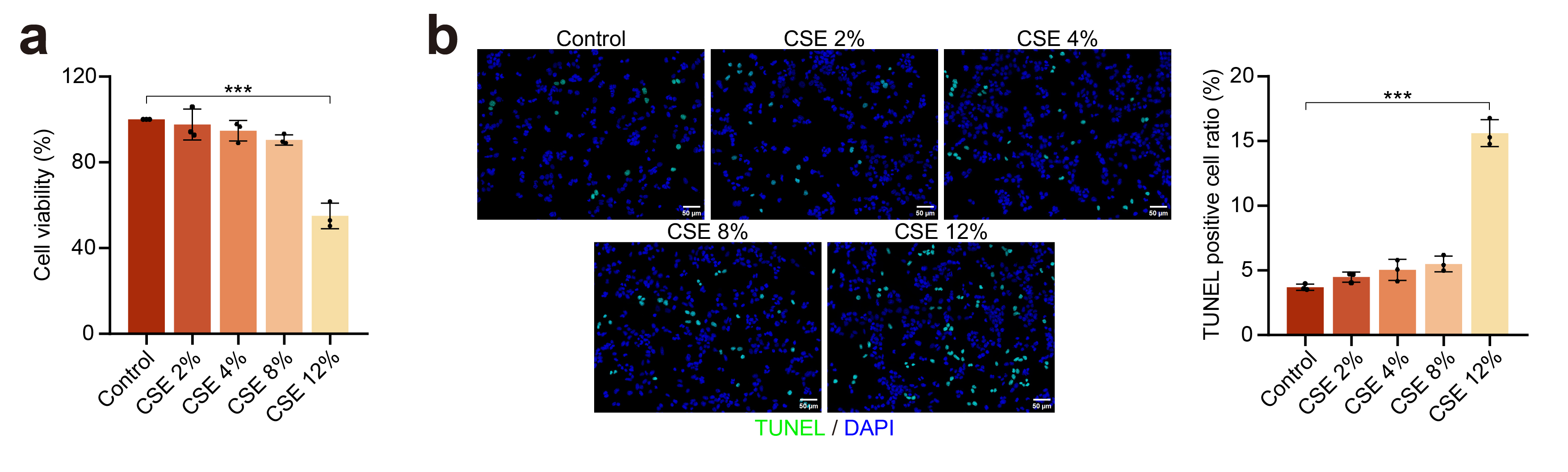


**Supplementary Figure S4. Screening of CSE intervention concentration. (a) Cell viability measured by CCK8 assay after treatment with increasing concentrations of CSE. (b) Cell apoptosis detected by TUNEL assay after treatment with increasing concentrations of CSE (magnification: ×200, scale bar = 50 μm). ***p< 0.001. Statistical significance was determined using one-way ANOVA.**


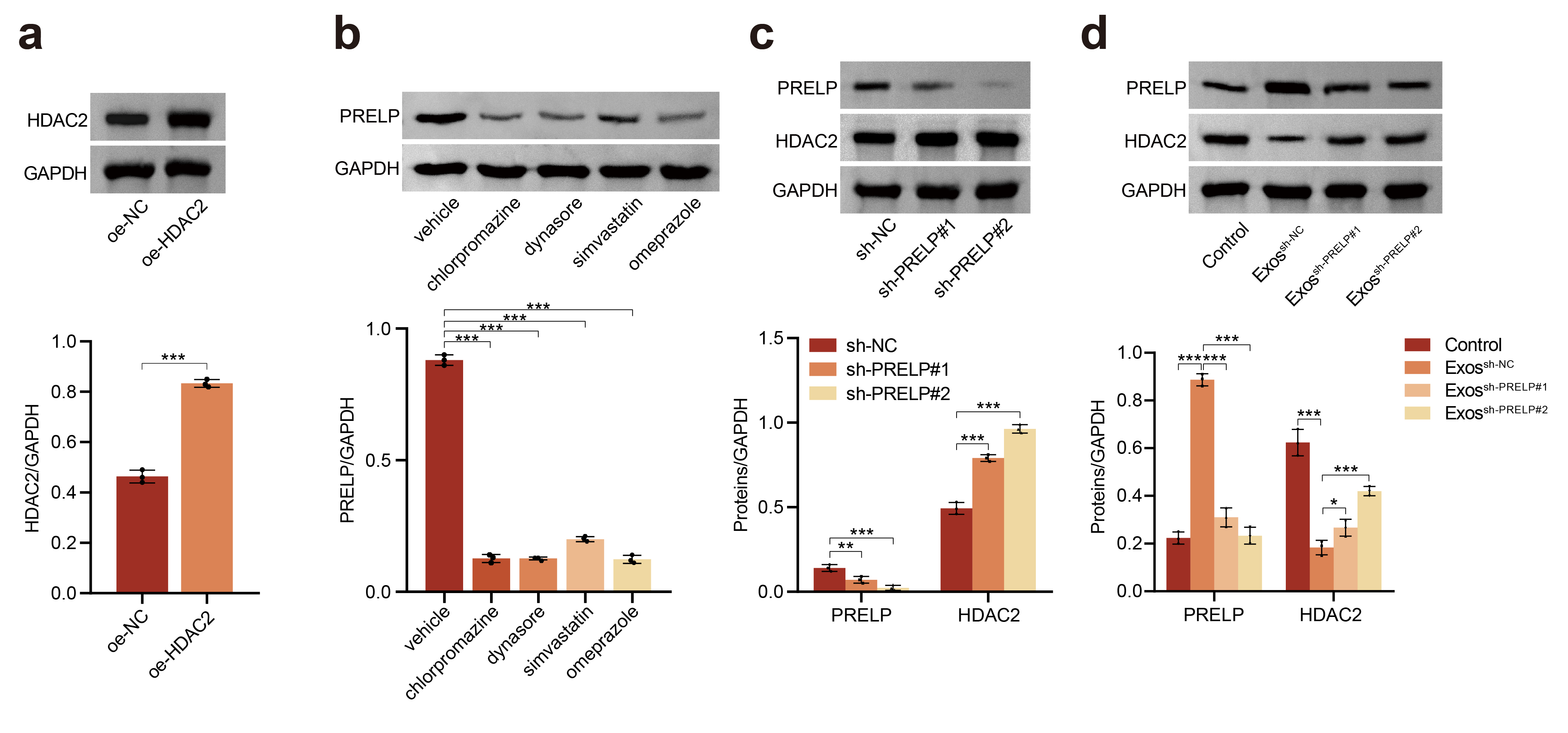


**Supplementary Figure S5. Validation of HDAC2 overexpression and analysis of PRELP-HDAC2 regulation.** (a) Validation of HDAC2 overexpression in C2C12 cells after oe-HDAC2 plasmid transfection by western blot. **(b) Western blot analysis of PRELP in C2C12 cells after endocytosis inhibitor treatment. (c) Western blot analysis of PRELP and HDAC2 expression after PRELP knockdown. (d) Western blot analysis of PRELP and HDAC2 in C2C12 cells treated with exosomes from PRELP-silenced MLE12 cells. n = 3; *p<0.05, **p<0.01, ***p< 0.001. Statistical significance was determined using an unpaired two-tailed Student's *t*-test (for a) or one-way ANOVA (for b, c, and d).**


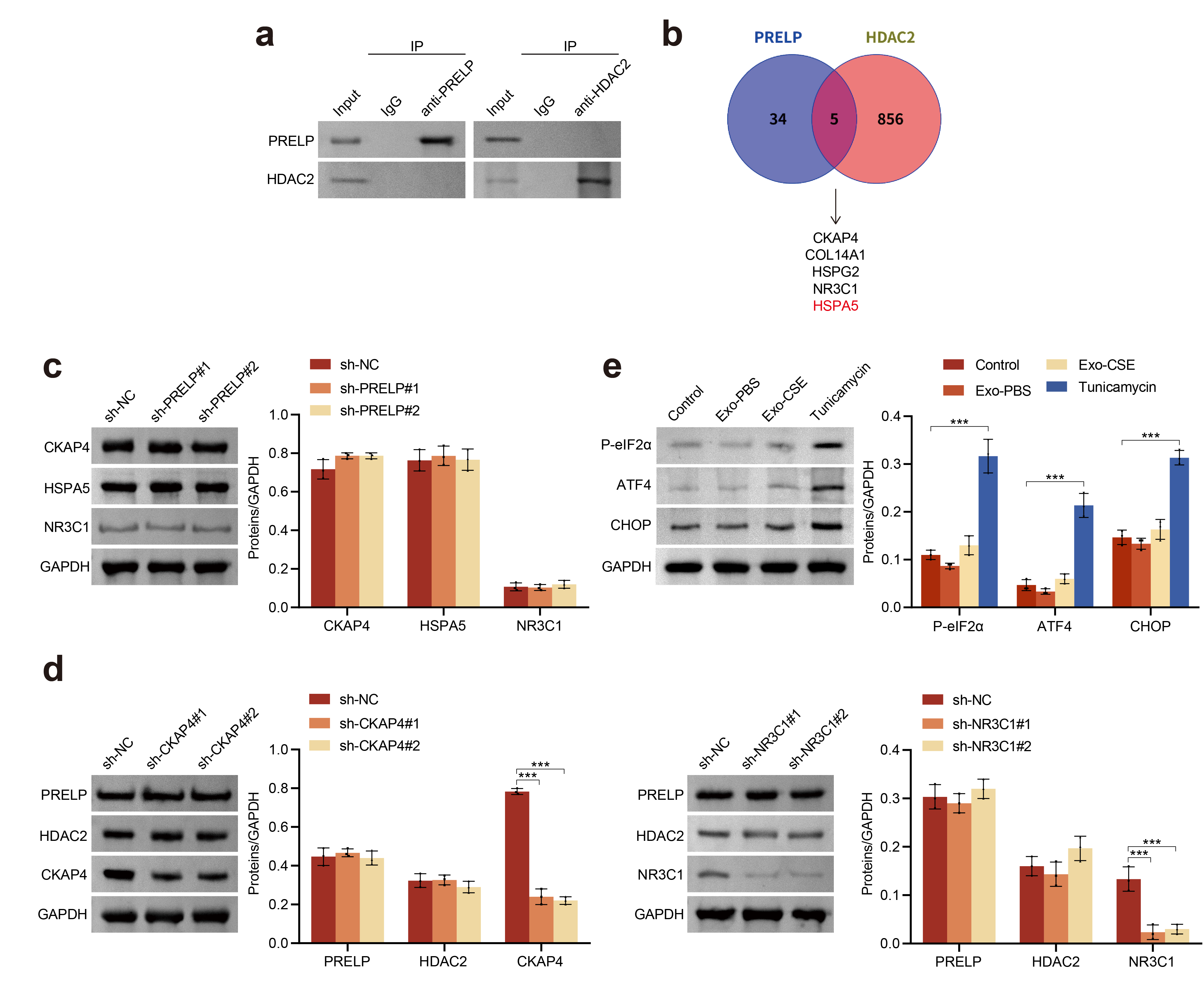


**Supplementary Figure S6. Validation of HDAC2 overexpression and analysis showing that CKAP4 and NR3C1 are not involved in PRELP-mediated regulation of HDAC2 in C2C12 cells.** **(a) Co-IP assay examining direct interaction between PRELP and HDAC2.** (**b**) Venn diagram showing predicted interacting proteins of PRELP and HDAC2 based on the BioGRID database. (**c**) Western blot analysis of CKAP4, HSPA5, and NR3C1 expression in C2C12 cells transfected with sh-NC or sh-PRELP. (**d**) Western blot analysis of PRELP, CKAP4, and HDAC2 in C2C12 cells transfected with sh-NC or sh-CKAP4, and of PRELP, NR3C1, and HDAC2 in C2C12 cells transfected with sh-NC or sh-NR3C1. (**e**) Western blot analysis of ER stress-related proteins p-eIF2α, ATF4, and CHOP. n = 3; ***p< 0.001. Statistical significance was determined using one-way ANOVA.


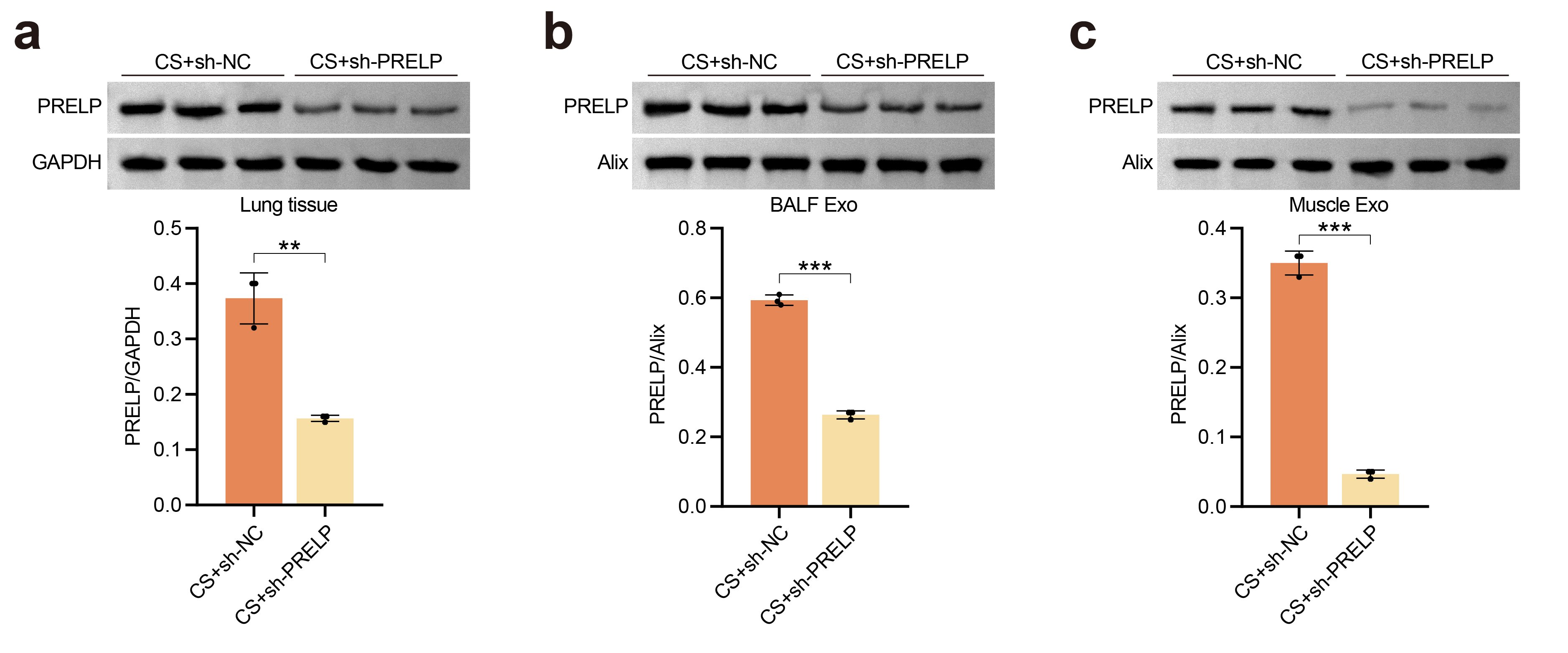


**Supplementary Figure S7. Validation of AAV carrying sh-PRELP knockdown efficiency. (a) Western blot detection of PRELP expression level in lung tissue.** (b) Western blot detection of PRELP expression level in lung-derived exosomes. (c) Western blot detection of PRELP expression level in skeletal muscle-derived exosomes. n = 3; **p<0.01, ***p< 0.001. Statistical significance was determined using an unpaired two-tailed Student's *t*-test.


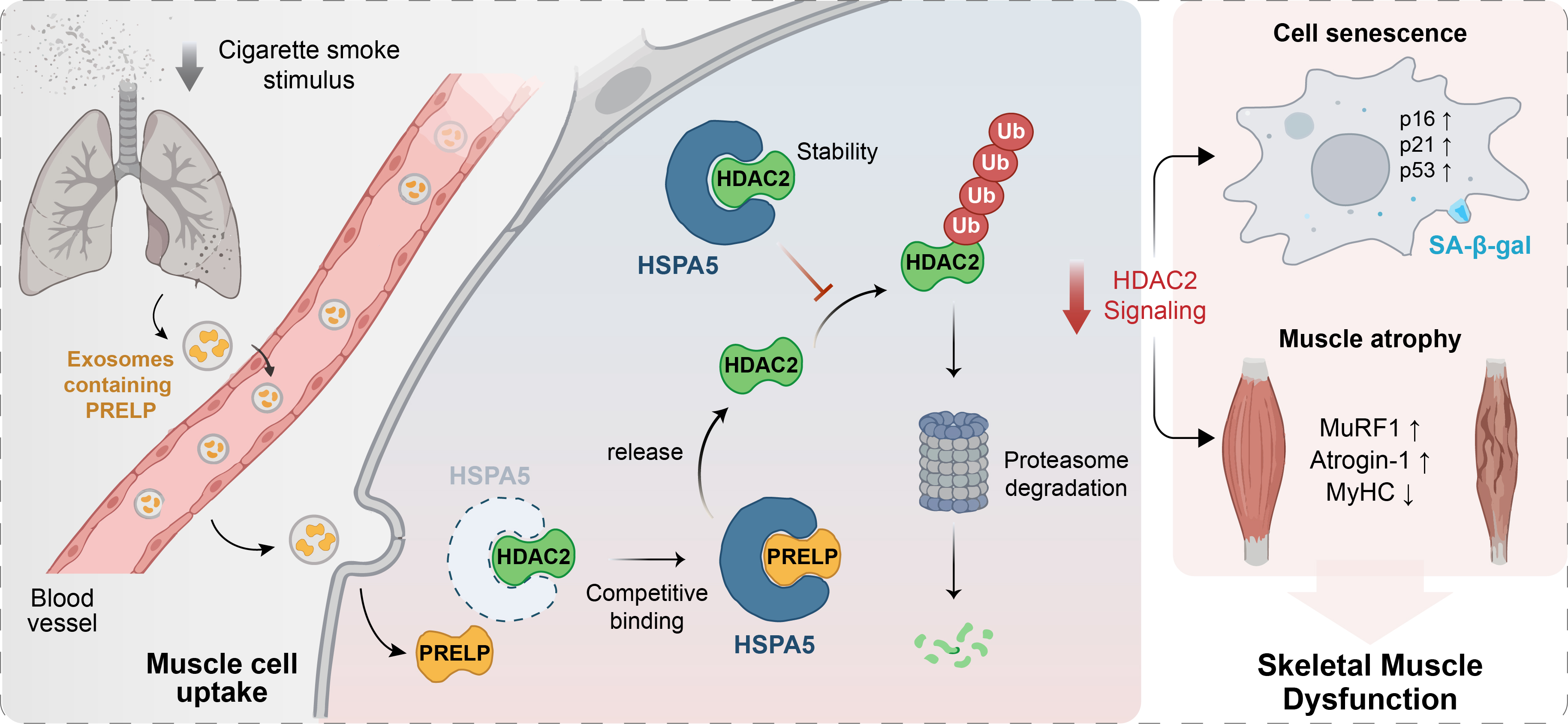


**Supplementary Figure S8. Schematic diagram. PRELP-enriched exosomes released by CS-exposed alveolar epithelial cells enter the systemic circulation and are taken up by skeletal muscle cells. Exosomal PRELP disrupts the HSPA5-HDAC2 interaction and promotes ubiquitin-mediated HDAC2 degradation, thereby inducing skeletal muscle cell senescence and muscle atrophy and ultimately contributing to skeletal muscle dysfunction.**

### Supplementary Tables

**Supplementary Table S1. The information on the antibody.**

| Name | Dilution rate | Cat. number | Source | Company | Country |
| --- | --- | --- | --- | --- | --- |
| MuRF1 | 1:3000 | ab183094 | Rabbit | Abcam | UK |
| Atrogin-1 | 1:15000 | 67172-1-Ig | Mouse | Proteintech | USA |
| SP-C | 1:1000 | 10774-1-AP | Rabbit | Proteintech | USA |
| MyHC | 1:20000 | 22281-1-AP | Rabbit | Proteintech | USA |
| MyOD | 1:1000 | ab133627 | Rabbit | Abcam | UK |
| MyOG | 1:10000 | 26762-1-AP | Rabbit | Proteintech | USA |
| P16 | 1:4000 | 10883-1-AP | Rabbit | Proteintech | USA |
| P21 | 1:2000 | 10355-1-AP | Rabbit | Proteintech | USA |
| P53 | 1:20000 | 10442-1-AP | Rabbit | Proteintech | USA |
| CD63 | 1:10000 | 67605-1-Ig | Mouse | Proteintech | USA |
| TSG101 | 1:8000 | 28283-1-AP | Rabbit | Proteintech | USA |
| GM130 | 1:5000 | ab52649 | Rabbit | Abcam | UK |
| HDAC2 | 1:10000 | 12922-3-AP | Rabbit | Proteintech | USA |
| PRELP | 1:1000 | 23783-1-AP | Rabbit | Proteintech | USA |
| CKAP4 | 1:40000 | 16686-1-AP | Rabbit | Proteintech | USA |
| HSPA5 | 1:6000 | 11587-1-AP | Rabbit | Proteintech | USA |
| NR3C1 | 1:10000 | 24050-1-AP | Rabbit | Proteintech | USA |
| p-eIF2α | 1:5000 | AP0692 | Rabbit | ABclonal | China |
| ATF4 | 1:500 | 10835-1-AP | Rabbit | Proteintech | USA |
| CHOP | 1:1000 | 15204-1-AP | Rabbit | Proteintech | USA |
| β-actin | 1:5000 | 66009-1-Ig | Mouse | Proteintech | USA |
| Alix | 1:10000 | 67715-1-Ig | Mouse | Proteintech | USA |
| HRP goat anti-mouse IgG | 1:5000 | SA00001-1 | / | Proteintech | USA |
| HRP goat anti-rabbit IgG | 1:6000 | SA00001-2 | / | Proteintech | USA |

**Supplementary Table S2. shRNA sequences.**

| Name | Sequence (5’-3’) |
| --- | --- |
| sh-NC | TTCTCCGAACGTGTCACGT |
| sh-PRELP#1 | CCGAATCCATTACCTTTACTT |
| sh-PRELP#2 | GTCTCACAACAAGATCAGCAA |
| sh-CKAP4#1 | CCAAGTCTATCAATGACAACA |
| sh-CKAP4#2 | CGAAACGAATGAGAATAACTT |
| sh-NR3C1#1 | CCCAGAGATGTTAGCTGAAAT |
| sh-NR3C1#2 | TTTGCTCCTGATCTGATTATT |
| sh-HSPA5#1 | CCGTACATTCAAGTTGATATT |
| sh-HSPA5#2 | GTAACAATCAAGGTCTATGAA |
| sh-HDAC2 | CCCAATGAGTTGCCATATAAT |

### Supplemental References

S1. Li C, Deng Z, Zheng G, Xie T, Wei X, Huo Z, Bai J. Resveratrol Prevents Skeletal Muscle Atrophy and Senescence via Regulation of Histone Deacetylase 2 in Cigarette Smoke-Induced Mice with Emphysema. J Inflamm Res. 2022;15:5425-37. doi:10.2147/JIR.S383180

S2. Guvatova ZG, Kobelyatskaya AA, Kudasheva ER, Pudova EA, Bulavkina EV, Churov AV, et al. Matrisome Transcriptome Dynamics during Tissue Aging. Life (Basel). 2024;14:593. doi:10.3390/life14050593

S3. Trajkovic K, Hsu C, Chiantia S, Rajendran L, Wenzel D, Wieland F, et al. Ceramide triggers budding of exosome vesicles into multivesicular endosomes. Science. 2008;319:1244-7. doi:10.1126/science.1153124

S4. Lee W, Chung WS, Hong KS, Huh J. Clinical usefulness of bronchoalveolar lavage cellular analysis and lymphocyte subsets in diffuse interstitial lung diseases. Ann Lab Med. 2015;35:220-5. doi:10.3343/alm.2015.35.2.220

S5. Rodriguez M, Silva J, Lopez-Alfonso A, Lopez-Muniz MB, Pena C, Dominguez G, et al. Different exosome cargo from plasma/bronchoalveolar lavage in non-small-cell lung cancer. Genes Chromosomes Cancer. 2014;53:713-24. doi:10.1002/gcc.22181

S6. Bengtsson E, Morgelin M, Sasaki T, Timpl R, Heinegard D, Aspberg A. The leucine-rich repeat protein PRELP binds perlecan and collagens and may function as a basement membrane anchor. J Biol Chem. 2002;277:15061-8. doi:10.1074/jbc.M108285200

S7. Bengtsson E, Aspberg A, Heinegard D, Sommarin Y, Spillmann D. The amino-terminal part of PRELP binds to heparin and heparan sulfate. J Biol Chem. 2000;275:40695-702. doi:10.1074/jbc.M007917200

S8. Gesteira TF, Verma S, Coulson-Thomas VJ. Small leucine rich proteoglycans: Biology, function and their therapeutic potential in the ocular surface. Ocul Surf. 2023;29:521-36. doi:10.1016/j.jtos.2023.06.013

S9. Chiavarina B, Ronca R, Otaka Y, Sutton RB, Rezzola S, Yokobori T, et al. Fibroblast-derived prolargin is a tumor suppressor in hepatocellular carcinoma. Oncogene. 2022;41:1410-20. doi:10.1038/s41388-021-02171-z

S10. Davaapil H, Hopkins J, Bonnin N, Papadaki V, Leung A, Kosuge H, et al. PRELP secreted from mural cells protects the function of blood brain barrier through regulation of endothelial cell-cell integrity. Front Cell Dev Biol. 2023;11:1147625. doi:10.3389/fcell.2023.1147625

S11. Nogueira AVB, Lopes MES, Marcantonio CC, Salmon CR, Mofatto LS, Deschner J, et al. Obesity Modifies the Proteomic Profile of the Periodontal Ligament. Int J Mol Sci. 2023;24:1003. doi:10.3390/ijms24021003

S12. Hanelova K, Raudenska M, Masarik M, Balvan J. Protein cargo in extracellular vesicles as the key mediator in the progression of cancer. Cell Commun Signal. 2024;22:25. doi:10.1186/s12964-023-01408-6

S13. Kosuge H, Nakakido M, Nagatoishi S, Fukuda T, Bando Y, Ohnuma SI, Tsumoto K. Proteomic identification and validation of novel interactions of the putative tumor suppressor PRELP with membrane proteins including IGFI-R and p75NTR. J Biol Chem. 2021;296:100278. doi:10.1016/j.jbc.2021.100278

S14. Lee AS. The ER chaperone and signaling regulator GRP78/BiP as a monitor of endoplasmic reticulum stress. Methods. 2005;35:373-81. doi:10.1016/j.ymeth.2004.10.010

S15. Ichhaporia VP, Kim J, Kavdia K, Vogel P, Horner L, Frase S, Hendershot LM. SIL1, the endoplasmic-reticulum-localized BiP co-chaperone, plays a crucial role in maintaining skeletal muscle proteostasis and physiology. Dis Model Mech. 2018;11:dmm033043. doi:10.1242/dmm.033043

S16. Leonard A, Grose V, Paton AW, Paton JC, Yule DI, Rahman A, Fazal F. Selective Inactivation of Intracellular BiP/GRP78 Attenuates Endothelial Inflammation and Permeability in Acute Lung Injury. Sci Rep. 2019;9:2096. doi:10.1038/s41598-018-38312-w

S17. Borok Z, Horie M, Flodby P, Wang H, Liu Y, Ganesh S, et al. Grp78 Loss in Epithelial Progenitors Reveals an Age-linked Role for Endoplasmic Reticulum Stress in Pulmonary Fibrosis. Am J Respir Crit Care Med. 2020;201:198-211. doi:10.1164/rccm.201902-0451OC

S18. Ito A, Hashimoto M, Tanihata J, Matsubayashi S, Sasaki R, Fujimoto S, et al. Involvement of Parkin-mediated mitophagy in the pathogenesis of chronic obstructive pulmonary disease-related sarcopenia. J Cachexia Sarcopenia Muscle. 2022;13:1864-82. doi:10.1002/jcsm.12988

S19. Deng M, Zhang Q, Yan L, Bian Y, Li R, Gao J, et al. Glycyl-l-histidyl-l-lysine-Cu(2+) rescues cigarette smoking-induced skeletal muscle dysfunction via a sirtuin 1-dependent pathway. J Cachexia Sarcopenia Muscle. 2023;14:1365-80. doi:10.1002/jcsm.13213

S20. Koide T, Mandai S, Kitaoka R, Matsuki H, Chiga M, Yamamoto K, et al. Circulating Extracellular Vesicle-Propagated microRNA Signature as a Vascular Calcification Factor in Chronic Kidney Disease. Circ Res. 2023;132:415-31. doi:10.1161/CIRCRESAHA.122.321939

S21. Liu Y, Zhou R, Guo Y, Hu B, Xie L, An Y, et al. Muscle-derived small extracellular vesicles induce liver fibrosis during overtraining. Cell Metab. 2025;37:824-41 e8. doi:10.1016/j.cmet.2024.12.005

S22. Behera J, Kelly KE, Voor MJ, Metreveli N, Tyagi SC, Tyagi N. Hydrogen Sulfide Promotes Bone Homeostasis by Balancing Inflammatory Cytokine Signaling in CBS-Deficient Mice through an Epigenetic Mechanism. Sci Rep. 2018;8:15226. doi:10.1038/s41598-018-33149-9

S23. Song J, Liu J, Cui C, Hu H, Zang N, Yang M, et al. Mesenchymal stromal cells ameliorate diabetes-induced muscle atrophy through exosomes by enhancing AMPK/ULK1-mediated autophagy. J Cachexia Sarcopenia Muscle. 2023;14:915-29. doi:10.1002/jcsm.13177

S24. Chhoy P, Brown CW, Amante JJ, Mercurio AM. Protocol for the separation of extracellular vesicles by ultracentrifugation from in vitro cell culture models. STAR Protoc. 2021;2:100303. doi:10.1016/j.xpro.2021.100303

S25. Sun Y, Zhang W, Li X. Induced pluripotent stem cell-derived mesenchymal stem cells deliver exogenous miR-105-5p via small extracellular vesicles to rejuvenate senescent nucleus pulposus cells and attenuate intervertebral disc degeneration. Stem Cell Res Ther. 2021;12:286. doi:10.1186/s13287-021-02362-1

S26. Liao L, Zheng Z, Deng M, Xu W, Zhang Q, Wang Z, et al. MG53 deficiency mediated skeletal muscle dysfunction in chronic obstructive pulmonary disease via impairing mitochondrial fission. Redox Biol. 2025;83:103663. doi:10.1016/j.redox.2025.103663
